# Interregional compensatory mechanisms of motor functioning in progressing preclinical neurodegeneration

**DOI:** 10.1016/j.neuroimage.2013.02.058

**Published:** 2013-07-15

**Authors:** Elisa Scheller, Ahmed Abdulkadir, Jessica Peter, Sarah J. Tabrizi, Richard S.J. Frackowiak, Stefan Klöppel

**Affiliations:** a Department of Psychiatry and Psychotherapy, University Medical Center Freiburg, Hauptstrasse 5, 79104 Freiburg, Germany; b Freiburg Brain Imaging Center, University Medical Center, University of Freiburg, Breisacher Str. 64, 79106 Freiburg, Germany; c Department of Psychology, Laboratory for Biological and Personality Psychology, University of Freiburg, Stefan-Meier-Str. 8, D-79104 Freiburg, Germany; d Department of Computer Science, University of Freiburg, Georges-Koehler-Allee, 79110 Freiburg, Germany; e Department of Neurology, University Medical Center Freiburg, Breisacher Str. 64, 79106 Freiburg, Germany; f UCL Institute of Neurology, University College London, Queen Square, London WC1N3BG, UK; g Département des Neurosciences Cliniques, CHUV, University of Lausanne, 1011 Lausanne, Switzerland

**Keywords:** BOLD, Blood Oxygen Level Dependent, cSMA, caudal supplementary motor area, DCM, Dynamic Causal Modelling, fMRI, functional Magnetic Resonance Imaging, GLM, General Linear Model, HC, healthy controls, lM1, left primary motor cortex, lPMd, left dorsal premotor cortex, lSPC, left superior parietal cortex, PET, positron emission tomography, preHD, preclinical Huntington's disease, rPMd, right dorsal premotor cortex, rSPC, right superior parietal cortex, TMS, Transcranial Magnetic Stimulation, VBM, Voxel-based Morphometry, fMRI, DCM, Neural reserve, Motor control, Pre-symptomatic Huntington's disease

## Abstract

Understanding brain reserve in preclinical stages of neurodegenerative disorders allows determination of which brain regions contribute to normal functioning despite accelerated neuronal loss. Besides the recruitment of additional regions, a reorganisation and shift of relevance between normally engaged regions are a suggested key mechanism. Thus, network analysis methods seem critical for investigation of changes in directed causal interactions between such candidate brain regions. To identify core compensatory regions, fifteen preclinical patients carrying the genetic mutation leading to Huntington's disease and twelve controls underwent fMRI scanning. They accomplished an auditory paced finger sequence tapping task, which challenged cognitive as well as executive aspects of motor functioning by varying speed and complexity of movements. To investigate causal interactions among brain regions a single Dynamic Causal Model (DCM) was constructed and fitted to the data from each subject. The DCM parameters were analysed using statistical methods to assess group differences in connectivity, and the relationship between connectivity patterns and predicted years to clinical onset was assessed in gene carriers.

In preclinical patients, we found indications for neural reserve mechanisms predominantly driven by bilateral dorsal premotor cortex, which increasingly activated superior parietal cortices the closer individuals were to estimated clinical onset. This compensatory mechanism was restricted to complex movements characterised by high cognitive demand. Additionally, we identified task-induced connectivity changes in both groups of subjects towards pre- and caudal supplementary motor areas, which were linked to either faster or more complex task conditions. Interestingly, coupling of dorsal premotor cortex and supplementary motor area was more negative in controls compared to gene mutation carriers. Furthermore, changes in the connectivity pattern of gene carriers allowed prediction of the years to estimated disease onset in individuals.

Our study characterises the connectivity pattern of core cortical regions maintaining motor function in relation to varying task demand. We identified connections of bilateral dorsal premotor cortex as critical for compensation as well as task-dependent recruitment of pre- and caudal supplementary motor area. The latter finding nicely mirrors a previously published general linear model-based analysis of the same data. Such knowledge about disease specific inter-regional effective connectivity may help identify foci for interventions based on transcranial magnetic stimulation designed to stimulate functioning and also to predict their impact on other regions in motor-associated networks.

## Highlights

• Connectivity of a motor network is altered in preclinical neurodegeneration.
• Dynamic Causal Modelling reveals task-dependent recruitment of pre- and caudal SMA.
• Connectivity of the dorsal premotor cortex reveals compensatory mechanisms.
• DCM allows prediction of years to clinical onset in preclinical patients.

## Introduction

Cognitive reserve (CR; Katzman, 1993; Stern, 2002; for a review see Valenzuela, 2008) is a concept to explain relatively preserved cognition in the face of neurodegeneration (Bartrés-Faz and Arenaza-Urquijo, 2011; Murray et al., 2011; Steffener et al., 2011). Passive CR is characterised in terms of brain size or number of neurons (e.g. Satz, 1993), whilst active CR refers to spontaneously variable reactions of the brain when faced with cognitive challenges. Neuroimaging can help to examine neural compensation (NC) as well as neural reserve (NR), which are subcomponents of active CR (Stern, 2009). NC describes the recruitment of additional brain areas to maintain performance, whilst NR reflects that impaired and non-impaired individuals use the same areas to maintain functioning, though to different levels of efficiency and capacity (Stern, 2009). NR is presumably instantiated by differential regional interactions (Seghier et al., 2010) shifting control from one set of regions to another (see below). Thus, one approach to investigate compensatory mechanisms in neurodegenerative diseases is to look at between-group as well as individual differences in NR (see e.g. Holtzer et al., 2009; Steffener et al., 2011). The assessment of compensatory mechanisms should preferably be undertaken in pre- or early clinical stages when therapeutic interventions are most likely to be effective. As such preclinical stages cannot easily be identified in the majority of neurodegenerative disorders, we chose to investigate Huntington's disease (HD), where preclinical stages can be identified with certainty and graded according to estimated proximity to symptom onset.

HD is a genetically caused hereditary neurodegenerative disease. As the exact location and nature of the genetic mutation are known (The Huntington's Disease Collaborative Research Group, 1993), it is possible to identify HD gene carriers decades before actual symptom onset. This clinical onset is defined by the presence of unequivocal motor symptoms (Beglinger et al., 2010; Walker, 2007). Therefore, patients without overt motor symptoms are described as ‘pre-manifest’ or ‘preHD’. Using a pre-manifest patient's current age and the degree of genetic mutation (i.e. the number of CAG trinucleotide repeats in the Huntingtin gene on chromosome four), the years to clinical onset (yto) can be estimated with a parametric survival model (Langbehn et al., 2004; Langbehn et al., 2010). The diagnostic status as well as the yto are used in neuroimaging studies to determine relationships with potential structural or functional imaging markers of the pre-manifest stage of disease (Feigin et al., 2006; Klöppel et al., 2008; Klöppel et al., 2009; Mühlau et al., 2007; Novak et al., 2012; Rosas et al., 2005; Scahill et al., 2013; Tabrizi et al., 2009; Tabrizi et al., 2011; Tabrizi et al., 2012; Wolf et al., 2007).

Previous studies focusing on compensatory mechanisms in motor functioning were conducted in the context of, among others, stroke (Grefkes et al., 2008b), preclinical Parkinson's disease (Buhmann et al., 2005) as well as mild to moderate HD and preHD (Bartenstein et al., 1997; Klöppel et al., 2009). Regarding HD, a supporting role of parietal motor related regions was first discussed in a PET experiment reported by Bartenstein et al. (1997). These parietal regions were more activated in HD patients than controls.

Nevertheless, Klöppel et al. (2009) stated that a simple shift in activation towards parietal regions might be too simplistic a view of the compensating mechanism and emphasised an additional role for the supplementary motor area (SMA), in which activations correlated with gene status, a finding well in line with those in patients with manifest HD (Gavazzi et al., 2007): Compared to healthy controls (HC), preHD activated caudal SMA during a finger tapping task to a greater extent in all movement conditions, and this activation increased with approaching clinical onset estimations. More complex finger movements led to even higher activations in pSMA in subjects further from predicted disease onset. Outside the SMA, the left superior parietal cortex (lSPC) showed reduced activation with increased movement complexity in preHD compared to HC, and in right SPC (rSPC), the preHD group showed greater activations in all but the most demanding conditions (Klöppel et al., 2009).

However, it is difficult to directly compare the studies of Bartenstein et al. (1997) and Klöppel et al. (2009), as the former authors investigated a sample of seven HD patients already exhibiting mild to moderate motor symptoms as opposed to the preHD group in Klöppel et al. (2009). The atrophy and loss of function were probably more severe in the mild to moderate HD group. Taken together the results suggest that the superior parietal cortices and pre- and caudal SMAs could contribute to compensatory motor mechanisms in preHD. An understanding of the interactions between cortical areas subtending compensation for the effects of neurodegeneration might help to shape therapeutic interventions (see e.g. Wang et al., 2011 for pharmacologically enhanced connectivity in the motor system). As an example, region specific interventions such as transcranial magnetic stimulation (TMS) can be applied most successfully to regions that are increasingly activated closer to disease onset. However, application to regions that exert an excitatory or inhibitory influence may also prove most useful (see e.g. Grefkes et al., 2010 for rTMS over M1) in the context of network function (see McIntyre and Hahn, 2010 for a review on deep brain stimulation).

A range of network analysis methods, such as Granger causality (Goebel et al., 2003; Granger, 1980) or Dynamic Causal Modelling (DCM; Friston et al., 2003) can be used to study effective connectivity (the influence one group of neurons has on another). Causal interactions between brain areas of interest can be studied and quantified; e.g. how does one region cause a change of activity in another, or how does a particular experimental manipulation influence the connectivity between two other regions. DCM has been successfully used to characterise motor function in healthy subjects and patients other than those suffering from HD (see e.g. Boudrias et al., 2012; Grefkes et al., 2008a; Grefkes et al., 2008b; Kasess et al., 2008; Rowe et al., 2010). In HD, classical functional connectivity analyses have been used in different cognitive domains (Wolf et al., 2008a, 2008b, 2012a, 2012b), but these correlational analyses do not allow inferences about causality to be made as correlated activity in two areas may be driven by a common third area (Stephan, 2004).

Therefore, DCM was chosen as our method to build on previous studies of motor function in preHD whilst overcoming the interpretational constraints of standard functional (as opposed to effective) connectivity analyses. The same data have been published before with a standard GLM analysis (Klöppel et al., 2009). We used DCM and a finger tapping task which leads to robust activations and probe functions that are specifically affected by HD (Bartenstein et al., 1997; Gavazzi et al., 2007; Lehéricy et al., 2006; Witt et al., 2008). The task does this by manipulating movement rate and complexity.

We analysed the nature of interactions between cortical regions of the motor system on the basis of DCM parameters, which constitute measures of connectivity, with two aims: First, as an increasing number of studies indicate the existence of neurodevelopmental and trait specific markers in HD (Lee et al., 2012; Marder and Mehler, 2012; Nopoulos et al., 2011), we compared such interactions in preHD with those of healthy controls (HC). Specifically, we expected to find more differences between preHD and HC with increasing task demands. Our second and key aim was to elucidate the development of NR as the time of expected symptom onset approached. Thus, we examined the relationship of inter-regional connectivity and yto in the preHD sample. As these subjects were in the pre-manifest stage, we considered reorganisation correlated with increasing neurodegeneration to be compensatory in nature. We expected to find alterations in connectivity between parietal and premotor and supplementary motor regions based on previous motor activation results.

## Materials and methods

### Participants

Fifteen pre-symptomatic gene mutation carriers (7 females, mean age 36.9 years, range 26–49) and twelve healthy controls (4 females, mean age 36.5 years, range 23–60), all right handed and matched according to age and sex were examined. A trained neurologist assessed all carriers with the Unified Huntington's Disease Rating Scale (UHDRS) to stage them. Pre-symptomatic participants covered a wide range of yto; these were computed as the number of years at which the predicted probability of clinical onset exceeds 0.6 (Langbehn et al., 2004); see Table 1 for demographic and clinical data. The local Ethics Committee approved the study and all participants gave written informed consent according to the Declaration of Helsinki. The current study is a reanalysis of the data that was published by Klöppel et al. (2009) as a standard GLM analysis. We extended on these previous findings by applying DCM.

### Experimental procedure and MRI scanning

Participants executed finger tapping sequences with the second to fifth fingers of their right hand paced by a metronome and delivered via headphones in the MRI scanner. Speed of pacing and complexity of the movements were varied systematically to challenge motor execution and higher motor control. Participants moved their fingers at a rate of 0.5 (slow sequence) or 2 Hz (fast sequence) and performed two types of sequences. The first ‘simple sequence’ consisted of regular unidirectional sequential button presses made with index, middle, ring and little fingers by all participants. The second ‘complex sequence’ was irregular without immediate repetitions, tapped with the same four fingers as the simple sequence but generated uniquely for each participant. Both sequences were trained prior to scanning until participants stated that they were comfortable performing them. A standard general linear model approach of these fMRI data has been reported previously (Klöppel et al., 2009) where a more detailed description of the task is provided.

Six types of experimental block of 20 s each and six blocks in total per condition (simple slow, simple fast, complex slow, complex fast, rest slow and rest fast) were presented in a pseudo-randomized order. During rest blocks, participants listened to 0.5 or 2 Hz clicks without pressing buttons. At the beginning of each block, instructions and numbers counting down from 3 to 0 in 3 s helped to prepare and start the tapping sequence on time.

Participants were scanned on a 1.5 T MRI system (Siemens Sonata; Erlangen, Germany) with the following scanning parameters: TR 3.6 s, TE 0.05 s, FOV 192 mm, flip angle 90 and 40 slices in descending order of 3 × 3 × 3 mm voxel size resulting in whole brain coverage. Total scanning time was around 15 min per participant. An additional T1-weighted MDEFT sequence (Deichmann et al., 2004) was acquired to exclude structural abnormalities unrelated to HD.

### Behavioural and fMRI data analysis

Error rates and mean and standard deviation of cue-response intervals were computed as reported in Klöppel et al. (2009). The initial processing of the data was also identical. In brief, realignment and spatial normalisation of volumes to a standard MNI template were followed by smoothing with an 8 mm full-width at half-maximum (FWHM) Gaussian kernel. Individual data were analysed in a General Linear Model (GLM; Friston et al., 1995) with separate regressors for each of the six experimental conditions and six regressors coding head movements.

### Effective network connectivity

#### Background of DCM

Studying cortical reorganisation in a network of known cortical areas in different experimental conditions is methodologically challenging. Simple correlations of the measured BOLD signal are insufficient to detect causalities (Stephan, 2004). This type of inference requires modelling of changing neuronal activity in different contexts from the recorded effects on the BOLD signal. DCMs describe the biophysical nature of directed interactions between brain areas (Friston et al., 2003) by incorporating two forward models, one at the neuronal and one at the haemodynamic level. A number of introductory articles are available (e.g. Friston, 2009; Seghier et al., 2010; Stephan et al., 2010) and the physiological basis of the approach is constantly being evaluated (Daunizeau et al., 2011; David et al., 2008).

At the neuronal level, the network with its nodes is expressed by a bilinear state equation, which contains three sets of model parameters predefined by the user: First, input parameters specify in which regions experimental stimuli (i.e. blocks with simple or complex finger presses) enter the model. Second, assumptions about the condition-independent connections between the nodes, which are not related to the task at hand, are specified. Those are often referred to as ‘fixed’ connections. In contrast, a third set of modulatory parameters expresses expected changes in connection strengths caused by these experimental conditions. The connection strength between regions is reported in Hz. A negative value is interpreted as decreased, a positive value as increased coupling from one region to another.

In addition to this neural model, the haemodynamic model (Stephan et al., 2007) contains parameters characterising blood flow and oxygenation change. The haemodynamic model ‘transforms’ the specifications of the neural model into a BOLD response to best match the modelled and actually measured BOLD responses with parameters that are physiologically plausible (Daunizeau et al., 2011; Stephan et al., 2007). Hence, knowing the experimental inputs as well as the output (i.e. the measured BOLD response) and viewing the brain as a dynamic input–state–output system, one can infer on underlying states such as regional causal interactions that remain hidden in conventional fMRI analyses. All model parameters are estimated in a Bayesian framework that combines existing a-priori knowledge with the actually measured data to generate a posterior probability distribution for each parameter.

#### Time series extraction

Time series were extracted for each participant according to anatomical and functional criteria as advised by Stephan et al. (2010), making sure to capture activity on an individual basis, whilst constraining individual peak activation to lie within a range from the group maximum. In a previous study, Klöppel et al. (2009) performed a ROI-based group analysis of the cortical motor system defining spheres centred on peak activations from the HC group in left primary motor cortex (lM1), pre-supplementary motor area (pSMA), caudal supplementary motor area (cSMA), left and right dorsal premotor cortices (lPMd, rPMd) as well as left and right superior parietal cortices (lSPC, rSPC) (Table 2).

First, we defined the same spheres with 10 mm radius as in the above-mentioned ROI analysis (centre MNI coordinates reported in Table 2) (Klöppel et al., 2009). Within each sphere, peak activations were located in every subject requiring a minimum threshold of p < .05 uncorrected (Stephan et al., 2010). Next, the first eigenvariate of the time series within 4 mm (i.e. half the size of the FWHM smoothing kernel used during preprocessing) of the subject-specific peak voxel was extracted and adjusted for effects of no interest (i.e. variance unexplained by the experimental conditions). Each time series was extracted from the contrast that should best reveal the activation of the respective region of interest: Time series from the cSMA were extracted from the contrast fast > slow condition, whereas time series for the remaining regions were extracted from the contrast complex > simple condition.

Before extraction, we ensured that no overlap of subject-specific spheres in neighbouring regions existed by checking the peak activation coordinates and surrounding spheres. An additional visual check of correct gyral location was performed in each region and corrections were applied if necessary.

#### Dynamic causal model specified

**Fig. 1:**
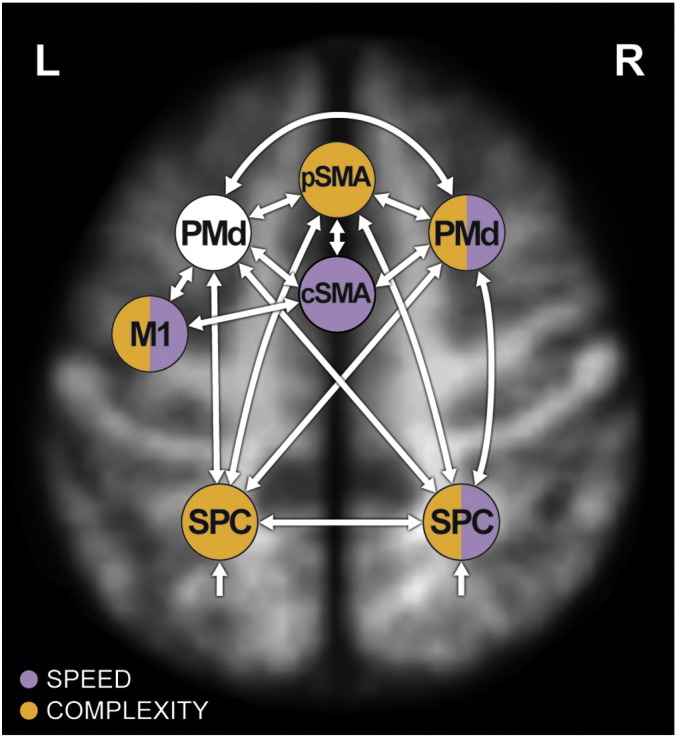
Dynamic Causal Model specified in each participant. White arrows represent condition-independent connections between regions of interest. Modulations of these connections by experimental factors, i.e. condition-dependent modulations, are colour-coded (purple for speed, orange for complexity modulation). The colour of the region represents the modulation by the respective experimental factor(s). All afferent connections from other regions towards the respective region are modulated. All specified model parameters can be reviewed in Supplement 3. For reasons of figure legibility, anatomical location of regions is approximated. The model is superimposed on a mean T1-weighted structural image of all participants, normalised to MNI space.

To investigate neural reserve in preHD, the time series of all seven cortical regions implicated in previous activation analyses were included in one DCM, constituting a network of cortical motor function: PSMA, cSMA, lPMd, rPMd, lSPC, rSPC and lM1 (Fig. 1). The slice timing option in DCM for fMRI was used (Kiebel et al., 2007) to account for regional acquisition time differences. Note that we explicitly chose to split the SMA in two sub-regions, as the pSMA is thought to be involved in more cognitively challenging conditions (see e.g. Nakamura et al., 1998; Rushworth et al., 2004) and is connected predominantly to prefrontal and parietal regions, whereas the caudal SMA represents the motoric executive part of the SMA being strongly interconnected with M1 as well as contributing to the corticospinal tract (Luppino et al., 1993; Nachev et al., 2008). rSPC and rPMd were differentially activated by the task and included in the model to account for potential contributions to NR from the ipsilateral hemisphere (Klöppel et al., 2009). Subsequently, interhemispheric connections between rSPC, lSPC, rPMd and lPMd as well as connections to pSMA (Geyer et al., 2000; Iacoboni, 2006: Mars et al., 2011; Narayana et al., 2012) were included. All connections between regions were specified bi-directionally, as it is physiologically plausible to assume a backward connection if a forward connection exists and vice versa (Kötter and Stephan, 2003; Zeki and Shipp, 1988).

We specified all experimental inputs (blocks of simple slow, simple fast, complex slow, complex fast, rest slow and rest fast conditions) to enter the model via associative sensory regions lSPC and rSPC which then distribute them to pSMA, lPMd and rPMd (see e.g. Koch and Rothwell, 2009). The experimental inputs entered the model as a single driving input, which we created as one regressor containing all experimental blocks.

We assumed no direct connection between pSMA and M1, but an indirect influence via lPMd and cSMA (Nachev et al., 2008). LM1 represents the ‘executive branch’ of the circuit specifying output to the corticospinal tract as participants performed the current task solely with their right hand.

Both complexity of the tapping sequence as well as speed of tapping execution were experimental factors used in the study of Klöppel et al. (2009). Both were therefore kept as distinct modulatory experimental inputs. On one hand, these modulatory inputs were specified in the DCM consistently with previous GLM analyses (Klöppel et al., 2009) — if a region exhibited differential activation between groups or interaction effects between conditions and groups in second level random effects analyses, or if activity in that region correlated with yto, we chose connections to this region to be modulated by the respective experimental factor (Fig. 1). On the other hand, we built on previous results that showed an interesting pattern of rPMd activity (Supplement 1) across conditions, such that HC subjects recruited this region more than preHD when task demands were more complex. With this analysis we additionally explored possible modulations of rPMd by speed and complexity. Finally, to disentangle potential influences of the more “cognitive” from more “executive” regions on lM1 (Nachev et al., 2008; Witt et al., 2008), we specified a modulation by speed on the afferent connection from cSMA (“executive function”) and by complexity on the connection from lPMd (“cognitive function” — Groppa et al., 2012; O'Shea et al., 2007b). Supplement 1 contains a more detailed description of the model specification process.

As HD is a neurodegenerative disease, it is important to mention the fact that potential atrophy of cortical regions is indirectly incorporated in a DCM. Parameters of a region-specific haemodynamic model are estimated within the DCM framework. Effects of atrophy on the respective neurovascular processes are incorporated in a subject-specific fashion (Stephan et al., 2007). In addition, we have done a voxel-based morphometry (VBM; Ashburner and Friston, 2000) analysis (data not reported in the manuscript) to illustrate which areas are affected by atrophy. When correcting for multiple comparisons (p < 0.05 FWE corrected), no areas showed reduced grey matter volume in preHD compared to HC. At a more liberal threshold of p < 0.001 uncorrected, striatal reductions became apparent. Thus, no area included in the DCM showed reduced grey matter volume in preHD compared to HC.

DCM specification and estimation was carried out with DCM10 in Statistical Parametric Mapping software (SPM8; Wellcome Trust Centre for Neuroimaging, http://www.fil.ion.ucl.ac.uk/spm).

#### Inference on model parameters: group analysis of DCM parameters

After model estimation in a Bayesian framework, the DCM of each participant was inspected with regard to the percentage of variance explained by using in-house MATLAB routines (version 7.14 R2012a; The Mathworks Inc., Natick, Massachusetts, USA). As a quality check we required that at least 10% of variance was explained. We then performed random effects inference on model parameter estimates using Wilcoxon signed rank tests with SPSS version 18.0 (PASW statistics) to assess whether the parameter estimates were significantly different from zero within groups (i.e. the existence of the parameters was confirmed by the model), and Wilcoxon rank sum tests to identify group differences.

#### Inference on model parameters: association of DCM parameters with years to onset

Rather than using all 65 predefined functional connecting and modulatory DCM parameters to predict yto, we applied principle component analysis (PCA) and varimax rotation with Kaiser's normalisation using SPSS. We aimed to reduce dimensionality of the data and sought to identify sets of parameters that would cluster together to facilitate interpretability. We decided to include both condition-independent and condition-dependent connections together in the PCA as in our understanding of compensation in preHD, it is well possible that a mixture of these connection strengths reflects compensatory mechanisms.

Having reduced data dimensionality, we used PCA group derived component values as predictors in a hierarchical multiple linear regression model to predict yto. This allowed us to relate DCM connection strengths to neurodegenerative processes. The age of preHD patients was included as a covariate with forced entry, because age is correlated with yto (Langbehn et al., 2004). The principal components (PCs) were then entered in a stepwise fashion until no further significant amounts of additional variance were explained. Subsequently, we examined the connection strengths with high loadings on the included PCs with regard to their individual correlations with yto.

An additional regression analysis was conducted with all standardised components derived from PCA to clarify if the combination of all PCs also predicts yto. Estimated yto was predicted with a regression using 10 rotated PCs and age as features. In a leave-one out cross-validation, the regression parameters were repetitively estimated with the data set containing all but one observation, and the error for each observation was estimated using the model that excluded the left-out observation. This avoided over-fitting, which would have resulted in over-optimistic regression performance that would naturally arise because the number of variables is very close to the number of observations. The PCA scores were computed only once in order to avoid fluctuations in the factors within cross-validation loops.

## Results

### Effective network connectivity: dynamic causal modelling

Time-series were extracted from each region of interest in each participant and included in individual DCMs (MNI coordinates provided in Inline Supplementary Table S2). Inspections of variance explained by the models and parameter estimability in three preHD patients led to their exclusion leaving 12 preHD patients and HC for further DCM analyses. Beforehand, we checked whether the exclusion biased any sample characteristics (Table 1), which was not the case.

Inline Supplementary Table S2

**Table S2:** MNI coordinates of individual peak voxel in each participant for volume of interest extraction (preHD N = 15; HC N = 12).

	pSMA			cSMA			lM1			lPMd			lSPC			rSPC			rPMd		
x	y	z	x	y	z	x	y	z	x	y	z	x	y	z	x	y	z	x	y	z
preHD																					
1	0	12	60	− 6	− 16	62	− 48	− 20	56	− 26	− 8	60	− 20	− 66	54	24	− 66	62	22	− 6	62
2	4	2	52	− 2	− 10	56	− 34	− 16	66	− 24	− 8	56	− 22	− 60	62	24	− 54	68	26	− 8	64
3	− 2	6	46	− 4	− 8	56	− 38	− 26	58	− 28	− 8	54	− 32	− 58	50	30	− 60	64	26	− 2	62
4	− 6	8	50	0	− 10	62	− 42	− 20	58	− 22	− 12	62	− 26	− 62	64	18	− 62	64	24	− 6	56
5	− 8	8	52	− 12	− 6	60	− 38	− 26	64	− 26	− 2	54	− 28	− 54	52	28	− 64	54	28	− 6	58
6	4	12	58	− 2	− 12	54	− 36	− 24	60	− 24	− 4	60	− 18	− 58	64	20	− 62	66	32	− 6	56
7	10	6	54	− 2	− 18	52	− 42	− 10	56	− 26	− 8	50	− 22	− 58	60	30	− 62	62	26	− 8	68
8	− 4	14	56	− 4	− 10	62	− 44	− 26	58	− 26	− 14	54	− 32	− 58	62	26	− 62	62	30	− 4	56
9	0	10	62	− 4	− 10	58	− 48	− 14	56	− 14	− 4	62	− 28	− 54	64	20	− 62	62	32	− 4	60
10	0	− 2	58	− 6	− 18	52	− 42	− 26	64	− 28	− 8	54	− 24	− 54	52	30	− 58	62	28	− 6	68
11	4	10	58	0	− 14	54	− 44	− 18	44	− 26	− 14	54	− 22	− 54	64	18	− 58	54	30	− 16	64
12	4	10	50	− 6	− 10	58	− 38	− 22	62	− 22	− 16	52	− 32	− 60	54	32	− 60	60	28	− 18	64
13	0	14	48	− 2	− 14	62	− 44	− 20	58	− 22	− 16	56	− 14	− 58	56	22	− 64	54	32	− 6	60
14	2	14	50	− 2	− 6	48	− 38	− 20	68	− 24	− 12	58	− 16	− 62	60	14	− 60	66	26	− 14	54
15	4	14	50	− 6	− 8	62	− 40	− 22	64	− 24	0	54	− 30	− 52	60	30	− 52	60	18	− 16	64

HC																					
1	6	2	56	0	− 12	54	− 38	− 22	62	− 14	− 6	62	− 24	− 64	56	22	− 62	64	28	− 8	64
2	− 8	4	46	− 14	− 14	56	− 38	− 22	56	− 24	− 8	60	− 24	− 62	64	22	− 62	58	28	− 12	58
3	8	10	56	− 6	− 8	58	− 42	− 12	58	− 22	− 12	58	− 20	− 56	64	26	− 52	68	18	− 8	64
4	0	14	18	0	− 16	56	− 38	− 26	64	− 18	0	54	− 22	− 60	60	22	− 64	62	28	− 16	56
5	2	10	60	− 6	− 6	54	− 38	− 26	60	− 24	− 8	62	− 32	− 54	60	30	− 64	58	28	− 4	64
6	0	8	50	− 2	− 18	58	− 40	− 14	66	− 22	2	46	− 24	− 58	66	28	− 54	68	28	− 18	64
7	0	4	54	− 2	− 18	50	− 38	− 12	58	− 24	− 6	52	− 24	− 64	60	22	− 66	58	24	− 4	52
8	− 4	10	54	− 2	− 10	56	− 38	− 18	60	− 26	− 8	58	− 24	− 54	− 64	32	− 60	60	26	− 2	68
9	8	6	56	− 2	− 10	52	− 40	− 22	62	− 26	− 8	58	− 20	− 62	64	26	− 52	68	28	− 6	68
10	0	10	50	0	− 6	52	− 36	− 24	62	− 18	0	60	− 26	− 58	62	18	− 60	58	30	− 8	60
11	− 4	12	48	− 4	− 14	50	− 46	− 22	54	− 12	− 6	60	− 24	− 66	52	28	− 58	64	30	− 10	68
12	− 4	10	50	− 8	− 6	62	− 40	− 8	60	− 26	− 10	58	− 22	− 64	56	26	− 64	64	30	− 10	66

Inline Supplementary Table S2

#### Inference on model parameters: group analysis of DCM parameters

**Fig. 2:**
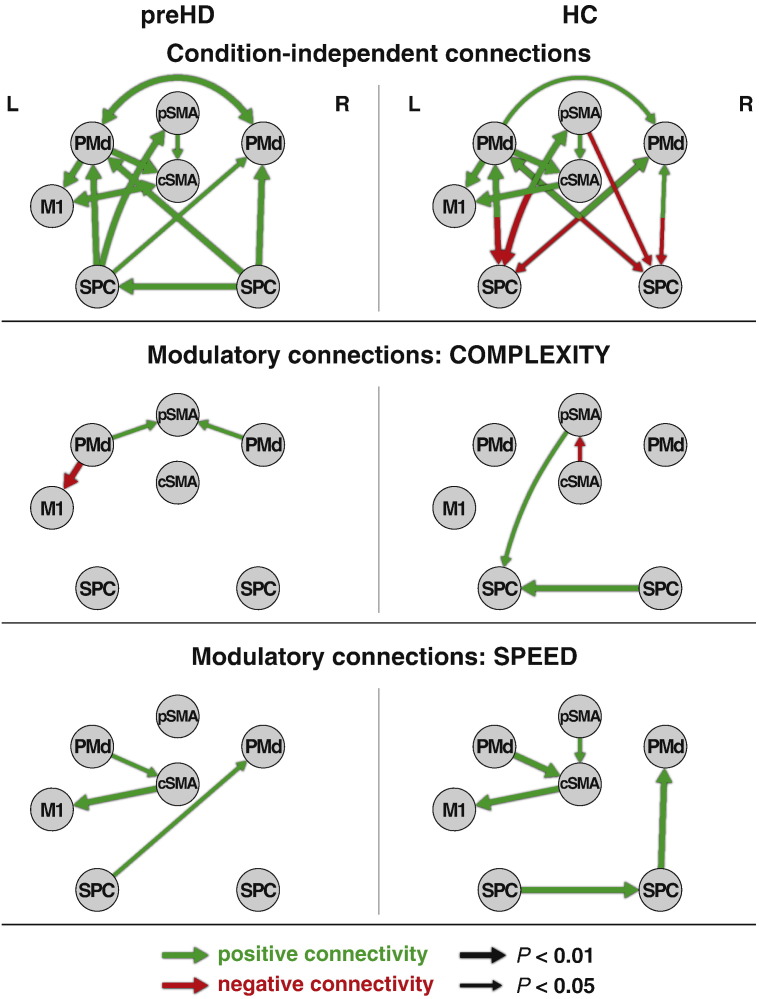
Condition-independent and modulatory DCM connection strengths in preHD (N = 12) and HC (N = 12) in a cortical network of motor functioning. Significant results from Wilcoxon rank sum tests are depicted. Arrow width represents the respective significance threshold. Numeric values of connection strengths can be derived from Supplementary Table S2.

Detailed descriptive statistics of all DCM parameters are provided in Inline Supplementary Table S3. The condition-independent and modulatory connections found to be significantly different from zero in Wilcoxon rank sum tests are depicted in Fig. 2 for both groups. Between group differences identified using Wilcoxon's signed rank test resulted from more negative connectivity in the HC group. Specifically, the condition-independent connection from pSMA to lSPC (p = 0.02) was stronger and more negative in HC compared to preHD and so were the effects of ‘complexity’ from cSMA to pSMA (p = 0.03) and from lPMd to pSMA (p = 0.03). The effect of ‘speed’ from rPMd to rSPC (p = 0.03) was more negative in HC as well, though not significantly different from zero in each group. Please note that these differences are mainly driven by the fact that these connections are significantly different from zero in HC, but not in preHD, where the mean group parameter estimate did not significantly differ from zero. These differences do not remain significant if Bonferroni corrections for multiple comparisons are applied (threshold p < 0.001 for condition-independent, p < 0.003 for ‘complexity’ modulatory connections and p < 0.004 for ‘speed’ modulatory connections define significance at p < 0.05 after correction).

Inline Supplementary Table S3

**Table S3:** Descriptive statistics of fixed and modulatory DCM parameter estimates and results from Wilcoxon signed rank tests to test whether the parameter is significantly different from zero within groups.

	preHD				HC		
DCM parameter	N	Minimum	Maximum	Median	Minimum	Maximum	Median
pSMA → pSMA, F	12	− 1.01	− .83	− .97^⁎⁎^	− 1.0	− .87	− .96^⁎⁎^
cSMA → pSMA, F	12	− .25	.19	− .01	− .18	.21	.03
lPMd → pSMA, F	12	− .23	.15	.02	− .09	.23	.05
lSPC → pSMA, F	12	− .12	.44	.13^⁎⁎^	− .085	.59	.24^⁎⁎^
rSPC → pSMA, F	12	.01	.79	.19	− .58	.43	.22
rPMd → pSMA, F	12	− .04	.20	.01	− .16	.35	.02
pSMA → cSMA. F	12	− .40	.65	.08^⁎^	− .12	.95	.21^⁎^
cSMA → cSMA, F	12	− 1.00	− .84	− .95^⁎⁎^	− 1.04	− .82	− .98^⁎⁎^
lM1 → cSMA, F	12	− .02	.38	.12^⁎⁎^	− .11	.23	.03
lPMd → cSMA, F	12	− .15	.75	.35^⁎⁎^	− .08	.80	.29^⁎⁎^
cSMA → lM1, F	12	.02	.48	.21^⁎⁎^	− .25	.92	.18^⁎⁎^
lM1 → lM1, F	12	− 1.02	− .83	− .98^⁎⁎^	− 1.03	− .73	− .96^⁎⁎^
lPMd → lM1, F	12	.02	1.02	.64^⁎⁎^	.17	.93	.63^⁎⁎^
pSMA → lPMd, F	12	− .29	.26	.00	− .20	.40	.05
cSMA → lPMd, F	12	− .23	.20	.03	− .18	.17	− .03
lM1 → lPMd, F	12	− .26	.11	.02	− .20	.13	− .04
lPMd → lPMd, F	12	− 1.07	− .82	− .96^⁎⁎^	− 1.04	− .83	− .95^⁎⁎^
lSPC → lPMd, F	12	.00	.84	.27^⁎⁎^	− .21	.85	.33^⁎⁎^
rSPC → lPMd, F	12	− .07	.88	.19^⁎⁎^	− .15	.58	.29^⁎⁎^
rPMd → lPMd, F	12	.02	.26	.10^⁎⁎^	− .19	.61	.07
pSMA → lSPC, F	12	− .16	.43	− .04	− .33	.00	− .12^⁎⁎^
lPMd → lSPC, F	12	− .36	.20	− .06	− .36	.04	− .20^⁎⁎^
lSPC → lSPC, F	12	− 1.03	− .97	− .99^⁎⁎^	− 1.06	− .93	− 1.01^⁎⁎^
rSPC → lSPC, F	12	− .02	.16	.05^⁎⁎^	− .14	.18	.04
rPMd → lSPC, F	12	− .38	.14	− .04	− .37	.03	− .13^⁎^
pSMA → rSPC, F	12	− .35	.24	− .06	− .27	.12	− .11^⁎^
lPMd → rSPC, F	12	− .53	.36	− .11	− .39	.29	− .06^⁎^
lSPC → rSPC, F	12	− .11	.20	− .00	− .14	.12	.00
rSPC → rSPC, F	12	− 1.05	− .88	− .99^⁎⁎^	− 1.03	− .91	− .98^⁎⁎^
rPMd → rSPC, F	12	− .32	.20	− .07	− .32	.05	− .15^⁎⁎^
pSMA → rPMd, F	12	− .19	.24	.01	− .27	.35	.07
lPMd → rPMd, F	12	− .06	.18	.04^⁎⁎^	− .05	.27	.05^⁎^
lSPC → rPMd, F	12	− .17	.33	.22^⁎^	− .15	.38	.26^⁎⁎^
rSPC → rPMd, F	12	− .00	.44	.18^⁎⁎^	− .07	.51	.19^⁎^
rPMd → rPMd, F	12	− 1.01	− .94	− .98^⁎⁎^	− 1.00	− .83	− .97^⁎⁎^
cSMA → pSMA, C	12	− .64	.73	.08	− 1.12	.23	− .29^⁎^
lPMd → pSMA, C	12	− .13	.30	.08^⁎^	− .67	.58	− .07
lSPC → pSMA, C	12	− .46	.99	.11	− .69	.91	.13
rSPC → pSMA, C	12	− .26	1.09	.19	− 1.16	.76	.22
rPMd → pSMA, C	12	− .44	.77	.19^⁎^	− .69	.60	− .01
lPMd → lM1, C	12	− 1.85	1.08	− .57^⁎^	− 1.60	1.21	− .23
pSMA → lSPC, C	12	− 2.2	.52	− .03	− .35	1.10	.18^⁎^
lPM → lSPC, C	12	− .33	.66	.01	− .37	.70	.02
rSPC → lSPC, C	12	− .55	.41	.09	− .04	1.37	.24^⁎⁎^
rPMd → lSPC, C	12	− .78	.51	− .01	− .80	.52	− .12
pSMA → rSPC, C	12	− 1.02	1.04	.01	− .70	.80	.18
lPMd → rSPC, C	12	− .59	1.05	.03	− .75	.74	.03
lSPC → rSPC, C	12	− .14	1.02	.08	− .35	.36	.24
rPMd → rSPC, C	12	− .91	.49	.02	− .78	.22	− .02
pSMA → rPMd, C	12	− .51	.65	.02	− .44	.28	− .02
lPMd → rPMd, C	12	− .37	.33	− .07	− .49	.41	.09
lSPC → rPMd, C	12	− 1.22	.40	− .07	− .49	1.09	.04
rSPC → rPMd, C	12	− .33	1.23	.09	− .45	.52	.01
pSMA → cSMA, S	12	− 1.06	.92	.00	− 1.31	2.30	.42^⁎^
lM1 → cSMA, S	12	− .58	.48	.01	− .69	.40	.05
lPMd → cSMA, S	12	− .13	1.12	.22^⁎^	− .27	1.11	.48^⁎⁎^
cSMA → M1, S	12	− .30	1.87	.65^⁎⁎^	− .00	2.32	.62^⁎⁎^
pSMA → rSPC, S	12	− 1.69	.35	− .01	− .90	1.37	− .02
lPMd → rSPC, S	12	− .37	.28	− .01	− .77	.27	− .07
lSPC → rSPC, S	12	− .16	.49	.10	− .23	.62	.28^⁎⁎^
rPMd → rSPC, S	12	− .37	.30	.02	− .52	1.26	− .21
pSMA → rPMd, S	12	− .45	.48	.01	− .56	.83	.07
lPMd → rPMd, S	12	− .57	.41	.00	− .46	.81	.11
lSPC → rPMd, S	12	− .31	.72	.28^⁎^	− .55	.28	.19
rSPC → rPMd, S	12	− .47	.63	.02	− .33	1.20	.35^⁎⁎^

Note: S = speed (experimental modulation), C = complexity (experimental modulation), F = fixed/condition independent connection.

Significance thresholds in Wilcoxon signed rank tests: ^⁎^ p < .05; ^⁎⁎^ p < .01.

Inline Supplementary Table S3

#### Dimensionality reduction of DCM parameters

The PCA with varimax rotation and Kaiser's normalisation identified ten PCs with eigenvalues higher than 1, which cumulatively explained 98.56% of total variance. An overview of factor loadings, the eigenvalues of the PCs, as well as percent variance explained after rotation can be found in Inline Supplementary Table S4. Note that we also included parameters which were not significantly different from zero in the PCA. We observed that parameter estimates varied substantially in preHD. Whist this led to non-significance across subjects, we reasoned that this variability may carry relevant information and thus decided to keep the respective parameters in the model.

Inline Supplementary Table S4

**Table S4:** Rotated component matrix of the PCA with fixed and modulatory DCM parameters of the preHD group with eigenvalues and percentage of explained variance.

DCM parameters	Component with loadings									
1	2	3	4	5	6	7	8	9	10
rPMd → rSPC, S	**.852**			.321		.238		− .322		
lSPC → rSPC, S	**− .849**	.166		− .286		− .287		− .188	.105	− .112
rPMd → rSPC, C	**.844**	− .122	− .153	.328			− .146		.168	.281
rSPC → pSMA, C	**− .842**			.112	− .172	.141	− .292	.194		.242
lPMd → lSPC, C	**.792**	− .307	− .185	− .340		.240		.230		
lSPC → rSPC, C	**− .701**	− .173	− .119	− .521				− .109	− .380	− .132
lPMd → rPMd, F	**.674**	− .284	.343		.297	.338		− .253		.269
lPMd → cSMA, F	**.633**					.344	.433		.135	.508
rPMd → lPMd, F	**− .631**	− .211	− .230		− .382	.555		− .169		
rSPC → rPMd, C	**− .610**	.168	− .371	.558	− .160	.208		− .208		
lPMd → rSPC, S	**.602**	.399	.167	− .122	.363	− .173	− .278	.108	.402	
rPMd → lSPC, S	**.549**		.338	.121	.125	− .247	.322	− .378	− .155	.407
pSMA → rSPC, C	.180	**.906**	.279	.239						
pSMA → lSPC, C		**.886**			− .254	− .335	− .115			
pSMA → rSPC, S		**.874**	.224		− .229	− .317	− .141			
pSMA → rPMd, S	− .233	**.817**	.228	− .239	.142	.169	− .119	.222	− .110	− .210
pSMA → cSMA, S	− .412	**.795**	.184	.252				− .209		− .187
pSMA → cSMA, F	.292	**.666**	− .168	.585	− .196					.200
pSMA → lSPC, F	.403	**− .549**	.342		.381	.506				− .123
lPMd → rSPC, F	.166		**.945**	− .223		− .111				
lPMd → lSPC, F		.226	**.933**		− .151	− .120				− .133
rPMd → rSPC, F	− .222	.352	**.874**		− .182					− .166
rPMd → lSPC, F		.394	**.792**	− .206	− .241	− .141	− .118		.130	− .246
pSMA → rSPC, F	.537		**.733**	.366						.133
pSMA → lPMd, F	− .174	.528	**.728**	− .105	− .208		− .187		− .262	
lSPC → rSPC, F	.471	− .113	**.656**		.179	.254	.417			.193
lSPC − lSPC, F		− .452	**.583**	.505			.238	− .266	− .245	
rPMd → pSMA, C	− .397	− .166	**− .521**	.381	.324	.480	.202	.122		
lPMd → pSMA, C	− .221	.107		**− .932**	− .168					.133
rPMd → pSMA, F	.292	.189	− .135	**.880**	− .204		− .177			
lPMd → rPMd, S		− .516	− .165	**− .785**	− .144	.109		.206		− .116
pSMA − pSMA, F			− .474	**.633**	− .130	.300		− .486		
rSPC → pSMA, F	− .341	.414	− .339	**.627**	− .103	.231	− .214	− .183		.102
cSMA → pSMA, F		− .168	.135	**− .621**	− .591	− .332	− .273	.117	.121	
rSPC → rPMd, S		− .206	− .290	**− .553**	.305	− .142	.164	.374	.417	.268
lPMd → rPMd, C	− .145			− .208	**− .875**		− .257	.168	− .116	− .227
rPMd → pSMA, F	.117		.366	.124	**− .872**		− .148	− .136		.147
rPMd → lSPC, C	.146	− .113	− .264		**.770**		− .190	.469	.191	
lPMd → pSMA, F		.405	.233	.277	**− .725**	− .142	.199	.240	.238	
lPMd → rSPC, C	.446	− .304	− .237	.111	**.701**	.316	− .105	.125	.113	
rSPC − rSPC, F	.177	.272	.399		**.618**	.399	.114	.342	.246	
lSPC → rPMd, F		− .157		.570	**.596**		− .156		.269	.420
rPMd − rPMd, F		− .266			.232	**.905**			.181	
cSMA − cSMA, F	.162	− .272	− .109		.124	**.879**	.250	− .183		
cSMA → lM1, F	− .190	.260	− .307	.313	− .232	**.723**		− .155	.268	.136
lSPC → pSMA, C	− .267		.270	− .216	.275	**− .659**	.280	− .263		− .228
lM1 → cSMA, F	.469	.191	.213		.159	**.655**	.263		.380	.155
pSMA → rPMd, C	− .305	.500		.435	.346	**.514**				− .260
rSPC → lSPC, F	.354	− .322	.269		.319	**.495**	− .206	.488	− .135	
rSPC → lSPC, C	− .299	− .185			− .107		**.904**	− .145		.106
lM1 → cSMA, S	− .175	− .111	− .486	.330			**− .747**	.105	.145	
cSMA → lPMd, F	− .308	.446	.218		− .215	− .155	**− .673**		− .340	
lPMd − lPMd, F		− .527	− .143			.409	**.572**	− .408	.149	.107
lM1 → lPMd, F	− .322	.326	.380		− .389	− .360	**− .560**		− .188	
lSPC → pSMA, F		.230	− .145	− .181				**.838**		
cSMA → pSMA, C	.210	− .230		− .316	.493	.328	− .122	**.639**		.137
lPMd → lM1, C	− .409		.134	− .279				**.613**	− .112	− .570
lPMd → cSMA, S		− .498		− .106	.573	.163	.135	**− .594**	.108	
rSPC → rPMd, F			.185	− .101	.343			− .188	**.808**	
lM1 − lM1, F					− .531	.319		− .218	**.718**	.146
rSPC → lPMd, F		.331	− .341	.160		.275	.309		**.693**	− .227
lSPC → rPMd, C		.171		− .388	.175	− .351	.243	− .223	**− .677**	− .290
lSPC → lPMd, F	.324	− .597	− .172	− .140	.132		.219			**.629**
lPMd → lM1, F	.262		− .436		.250	.237	.288	− .273	.330	**.597**
cSMA → lM1, S	.333	− .408	− .380	.439		.373				**.448**
Eigenvalues before rotation (after rotation)	16.22 (9.54)	10.28 (8.94)	9.90 (8.47)	6.44 (7.51)	5.81 (7.27)	4.85 (6.81)	4.13 (4.34)	2.78 (4.33)	1.96 (3.82)	1.68 (3.05)
% variance explained before rotation (after rotation)	24.96 (14.67)	15.82 (13.76)	15.24 (13.03)	9.91 (11.56)	8.93 (11.19)	7.45 (10.48)	6.36 (6.68)	4.27 (6.66)	3.02 (5.87)	2.59 (4.68)

Note: S = speed (experimental modulation), C = complexity (experimental modulation), F = fixed/condition-independent connection.

Inline Supplementary Table S4

#### Prediction of years to onset with DCM parameters and assessment of neuronal reserve

Multiple linear regression analyses identified relationships between the extracted PCs and predicted yto. A hierarchical multiple linear regression analysis with age as a covariate included PC 1 (Table 3), which explained an additional 27.7% of variance. The stepwise inclusion of further PCs in the model led to insignificant changes in R^2^ and hence no further PCs were added. The Durbin–Watson statistic (coefficient = 2.27), checks for homoscedasticity as well as non-multicolinearity, confirmed the validity of the model. A negative bivariate correlation with yto (r = − 0.73, p = 0.007) indicated a stronger expression of the component closer to expected disease onset.

**Fig. 3:**
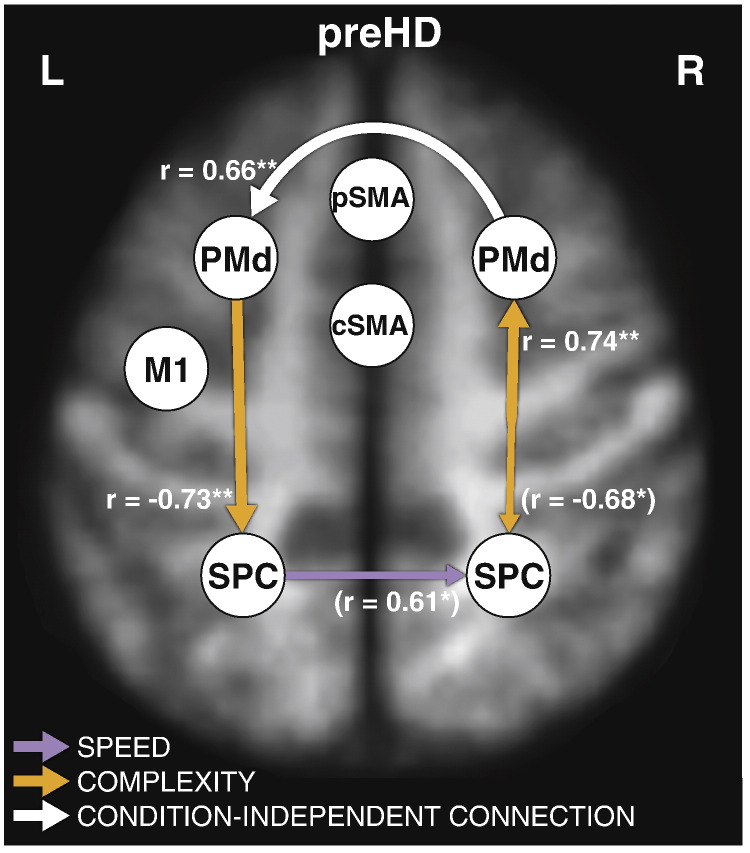
Connection strengths correlating with yto in preHD. Correlation coefficients in brackets do not explain an additionally significant amount of variance after controlling for age. Note that positive values of r indicate decreased coupling and negative values of r indicate increased coupling with nearing disease onset The model is superimposed on a mean T1-weighted structural image of all participants with anatomical location of regions approximated, normalised to MNI space. * = p < 0.05; ** = p < 0.01.

PC 1 contained high loadings of twelve DCM parameters involving connections of bilateral SPC and PMd (Inline Supplementary Table S4). Post-hoc examination revealed bivariate correlations of the yto with five of these connection strengths (Fig. 3; Inline Supplementary Table S3). This number reduced to three after including age as a covariate (see Fig. 3, correlation coefficients without brackets). Of note, a positive correlation between yto and a parameter effectively indicate that the coupling decreases significantly with approaching disease onset, after correcting for age effects: Here, the condition-independent causal influence of rPMd on the lPMd decreases as does the ‘complexity’ modulatory connection from rSPC to rPMd. In contrast, a negative correlation between yto and a parameter indicates that the coupling increases significantly with approaching disease onset. In our model, (Fig. 3), complexity associated modulatory connectivity from lPMd to lSPC increases when a patient is closer to disease onset.

**Fig. 4:**
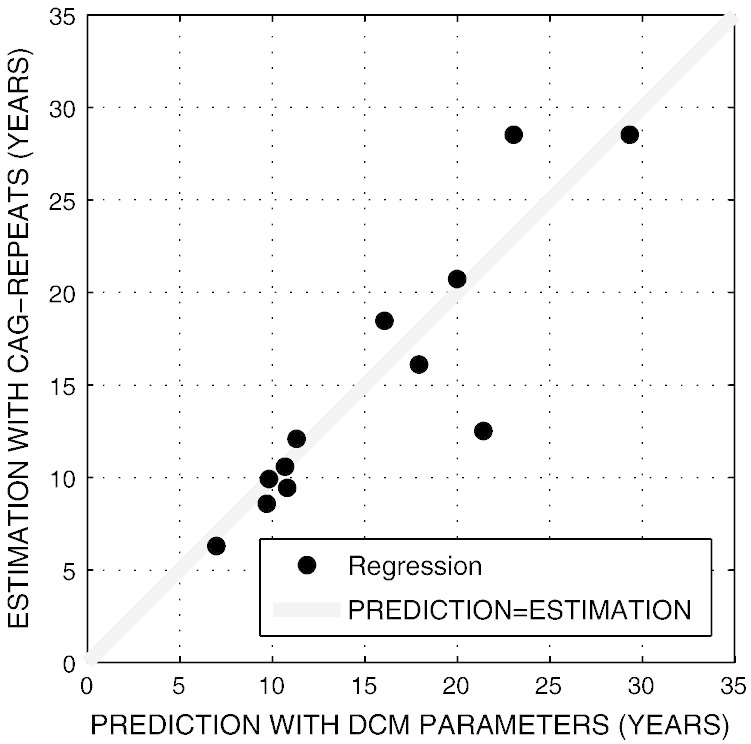
Linear Regression analysis of all condition-independent and modulatory DCM connection strengths to predict yto in preHD. X-coordinates show the prediction with DCM parameters, whilst Y-coordinates represent estimation with the model by Langbehn et al. (2004). The angle bisector depicts perfect match of prediction and estimation.

The second linear regression with 10 PCs and age as features resulted in a Linear Regression Correlation of r = 0.90 (p = 0.01%), with a root mean squared error of 3.2 years (Fig. 4).

## Discussion

We aimed to investigate preHD neural reserve mechanisms with a DCM analysis of cortical motor function. Our results indicate a crucial role of PMd which adds to that of the pre- and caudal SMA demonstrated here and in an earlier analysis using standard activation based methods (Klöppel et al., 2009).

### Connectivity analysis

The DCM analysis confirms motor system connectivity characteristics similar in strength and polarity compared to previous studies (see e.g. Grefkes et al., 2008a). Condition-independent connectivity was widespread within the network and included anterior–posterior inhibitory connectivity towards SPC in HC (Boudrias et al., 2012), which did not reach significance in preHD. Significant modulatory connections were found for the experimental factors speed and complexity, involving connections towards cSMA in the former and pSMA in the latter in both subject groups (Fig. 2).

### Between-group analysis of DCM parameters

Although not statistically significant after correction for multiple comparisons, we observed more positive condition-independent and modulatory connectivities between several regions in preHD compared to controls (Fig. 2, Inline Supplementary Table S3): A decline in inhibitory condition-independent connections points to a reduction of inhibition from supplementary and premotor regions towards bilateral SPC in preHD. This is well in line with the task-dependent correlations of PMd–SPC connections discussed below, which gain strength with approaching disease onset, and also with findings of decreased effective inhibitory connectivity in ageing (Boudrias et al., 2012).

The between-group comparison reveals a reorganisation of the motor system in the pre-manifest state with a task performed equally well by both subject groups (Klöppel et al., 2009). Higher task demands are likely to reveal even stronger disease effects as shown by Wolf et al. (2008b), who found aberrant fronto-striatal coupling in preHD only under high working memory load. Of note, our sample is relatively far from clinical disease onset.

DCM now allowed us to identify the network characteristics underlying differential recruitment of the SMA observed in the preHD group in a previous analysis of the same data (Klöppel et al., 2009). Greater effects of ‘complexity’ from cSMA to pSMA and from lPMd to pSMA in the preHD group, and increased condition-independent connections from pSMA to lSPC, may well be a reflection of those observations. In Klöppel et al. (2009), we reported greater activation of cSMA in preHD and correlations of pSMA and cSMA with approaching disease onset. An equivalent of these findings may be the tighter couplings of DCM parameters connected to SMA in preHD compared to controls.

### Prediction of yto with DCM parameters and assessment of potential NR

Our results from two multiple regression models show an association between connection strengths and yto. In a hierarchical multiple linear regression model with age as a covariate, one PC contributed significantly to the prediction of yto. On inspection, and after controlling for age, we found three significant correlations of connection strengths loading high on this PC, involving PMd and SPC bilaterally: First, the positive correlations of complexity-associated modulation from rSPC to rPMd with yto suggest that rSPC exerts less influence on rPMd with approaching disease onset, when the task becomes cognitively challenging. Second, the positive correlation of the fixed connection from rPMd to lPMd suggests an attenuated impact of rPMd on lPMd with approaching clinical onset independently of task demands. This could point to declining interhemispheric influences from rPMd to lPMd or – as the disease progresses – an inhibition of lPMd.

Third, and most important in relation to NR, is the increasing complexity-associated modulation of lPMd to lSPC connectivity with approaching disease onset. Of note, we found a similar increase in connectivity in corresponding areas in the right hemisphere, which did not remain significant after controlling for age (see e.g. Tanaka and Watanabe, 2011, for findings on compensation of ipsilateral sensorimotor regions to maintain motor functioning). Connections from PMd to SPC are part of widely connected parieto-frontal circuits (Iacoboni, 2006; Matelli and Luppino, 2001). A key role of the lPMd in motor control has been suggested previously (Schluter et al., 1998). In the context of HD and preHD, SPC has been discussed in terms of a parietal shift (Bartenstein et al., 1997) and parietal interaction effects (Klöppel et al., 2009) reported in activation analyses. Our findings suggest that this shift is expressed as enhanced positive effective connectivity from PMd to SPC, whilst connectivity in the other direction declines (Fig. 3). Hence, with greater proximity to symptom onset, the SPC is more strongly recruited by PMd to ensure motor functioning in periods of cognitive challenge (Rizzolatti et al., 1997; Rushworth et al., 2003). Again, a role of parietal cortex in compensation has recently been discussed by Wolf et al. (2012b), who found the default mode network, which includes the inferior parietal lobe, to be functionally persistent in preHD. Furthermore, it interacts with PMd for reaching movements as revealed by TMS (Busan et al., 2009), whilst PMd overall exerts a steering role in the guidance of hand movements (Groppa et al., 2012; Koch et al., 2006; O'Shea et al., 2007a; Schluter et al., 1998). Taken together, the interplay of parietal and frontal motor areas during action execution is highly variable and task-dependent (Koch and Rothwell, 2009; Rounis et al., 2005), which is confirmed by the identification of PMd–SPC correlations solely in complex task conditions. These connectivity results therefore complement those from the standard activation analyses.

A second linear regression analysis confirmed the predictive power of all derived connection strengths. Of note, we compared the predictions of the biophysical DCM to predictions of the genetically motivated model by Langbehn et al. (2004) without knowing the true value of yto. To address the predictive power of any model, a longitudinal design including the individual conversion times would be needed.

### Limitations

A relatively small sample may explain the absence of significance between group differences after correction for multiple comparisons. Nevertheless, the approach was based on a robustly activating experimental paradigm and we obtained precise predictions of the yto with DCM parameters in such a small sample (Friston, 2012). Thus, confidence in the connectivity strengths derived from our network analysis method is enhanced. We emphasise that results from connectivity analyses do not always reveal effects analogous to activation analyses (see e.g. Wolf et al., 2012a) and should be interpreted and viewed as a different approach to the same data but not as another means of drawing the same conclusions (Fox and Friston, 2012; Friston, 2009). Furthermore, we realize that the DCM parameters are conditional on the model tested and that we cannot interpret model structure itself in the current study (Daunizeau et al., 2011; Seghier et al., 2010); here, the scope was inference on model parameters. For inference on model structure, a Bayesian Model Selection analysis would have been needed (Stephan et al., 2009; Stephan et al., 2010; Supplement 1) to determine which of the investigated connections best explain the data. In summary, connectivity-related research questions lead to findings involving very similar regions to those identified in activation analyses. However the results reveal the nature of other additional relationships between the regions in the context of our experimental groups and the tasks at hand.

### Conclusions and implications

Taken together, the current study indicates that the potential NR for motor function in a preHD sample is provided by differential support by pre- and caudal SMA during task-induced challenges driven by PMd, and an important role for the positive directed interaction from bilateral PMd to SPC, which gains strength with proximity to clinical onset.

As mentioned in the introduction, HD comprises a combination of developmental and state markers. To identify state markers, we performed a comparison against a control group. Additional correlation analyses in the HD group with yto were performed to focus on developmental trait markers with the limitation of cross-sectional design. As gene-mutation carriers showed matched performance compared to controls and because we focus on changes correlating with yto, the observed changes are likely to be compensatory in nature.

Identifying potential NR mechanisms and explanations for the neural underpinnings of CR may suggest therapeutic interventions to cushion and delay cognitive decline in various disorders (see e.g. discussions in Buhmann et al., 2005; Eickhoff et al., 2008; Grefkes et al., 2008b). Such interventions, designed to stimulate CR, have already been successfully applied in animal models (Nithianantharajah and Hannan, 2011; Nithianantharajah et al., 2009; Valenzuela, 2008; Wood et al., 2011). In humans, interventions exploiting connectivity analyses could involve locally exciting TMS or tDCS protocols (e.g. Grefkes et al., 2010), which have shown promising relationships with DCM parameters in ageing and healthy controls (Boudrias et al., 2012; Sarfeld et al., 2012). Such protocols should focus on regions predominantly modulating others that subtend a specific cognitive domain so as to increase a specific cognitive reserve.

The following are the supplementary data related to this article.

Supplement 1 Dynamic Causal Modelling: motivation of model specification.

## Funding

Data acquisition was supported by the Wellcome Trust (075696 2/04/2 to R.S.J.F., S.J.T. and John Ashburner).

## Conflict of interest

The authors declare no conflict of interest.

## Figures and Tables

**Table 1:** Demographics of gene carriers (preHD) and controls (HC) reported with median and range.

	HC	preHD
Number of participants	12	15
Female/male	4/8	7/8
Age	32.5 (23: 60)	37 (26: 54)
Number of CAG repeats	NA	42 (39: 47)
UHDRS motor score	NA	2 (0: 17)
Years to 60% probability of clinical onset	NA	12.51 (6.3: 35.4)

**Table 2:** Regions of interest in fMRI analysis and MNI peak coordinates of spheres used to search for individual peak activation to extract volumes of interest in DCM analysis.

Region	Hemisphere	x	y	z
Caudal SMA	L	− 6	− 10	54
Primary motor cortex	L	− 40	− 18	60
Pre SMA		0	6	54
Dorsal premotor cortex	L	− 24	0	54
Dorsal premotor cortex	R	26	− 6	52
Superior parietal cortex	L	− 22	− 68	58
Superior parietal cortex	R	22	− 66	60

**Table 3:** Hierarchical multiple linear regression model with age as a covariate and stepwise inclusion of connectivity-representing components from PCA to predict yto.

Model	Included variables	B	SE B	Beta	R^2^	Adjusted R^2^
1	Constant	37.58	7.37			
Age	− .56	.18	− .70⁎	.49⁎	.32
2	Constant	31.75	5.52			
Age	− .41	.13	− .52⁎		
Component 1	− 4.18	1.27	− .56⁎⁎	.77⁎⁎	.61

Note: B = unstandardized regression coefficients; SE B = standard error of B; Beta = standardised regression coefficients.

R^2^ was adjusted according to Stein's formula.

⁎ p < 0.05.

⁎⁎ p < 0.01.

## References

[bb0005] Ashburner J., Friston K.J. (2000). Voxel-based morphometry—the methods. Neuroimage.

[bb0010] Bartenstein P., Weindl A., Spiegel S., Boecker H., Wenzel R., Ceballos-Baumann A.O. (1997). Central motor processing in Huntington's disease. A PET study. Brain.

[bb0015] Bartrés-Faz D., Arenaza-Urquijo E. (2011). Structural and functional imaging correlates of cognitive and brain reserve hypotheses in healthy and pathological aging. Brain Topogr..

[bb0020] Beglinger L.J., O'Rourke J.J.F., Wang C., Langbehn D.R., Duff K., Paulsen J.S. (2010). Earliest functional declines in Huntington disease. Psychiatry Res..

[bb0030] Boudrias M.-H., Gonçalves C.S., Penny W.D., Park C., Rossiter H.E., Talelli P. (2012). Age-related changes in causal interactions between cortical motor regions during hand grip. Neuroimage.

[bb9000] Buhmann C., Binkofski F., Klein C., Büchel C., Eimeren T. van, Erdmann C., Hedrich K., Kasten M., Hagenah J., Deuschl G., Pramstaller P.P., Siebner H.R. (2005). Motor reorganization in asymptomatic carriers of a single mutant Parkin allele: a human model for presymptomatic parkinsonism. Brain.

[bb0040] Busan P., Barbera C., Semenic M., Monti F., Pizzolato G., Pelamatti G. (2009). Effect of Transcranial Magnetic Stimulation (TMS) on parietal and premotor cortex during planning of reaching movements. PLoS One.

[bb0045] Daunizeau J., David O., Stephan K.E. (2011). Dynamic causal modelling: a critical review of the biophysical and statistical foundations. Neuroimage.

[bb0050] David O., Guillemain I., Saillet S., Reyt S., Deransart C., Segebarth C. (2008). Identifying neural drivers with functional MRI: an electrophysiological validation. PLoS Biol..

[bb0055] Deichmann R., Schwarzbauer C., Turner R. (2004). Optimisation of the 3D MDEFT sequence for anatomical brain imaging: technical implications at 1.5 and 3 T. Neuroimage.

[bb0060] Eickhoff S.B., Dafotakis M., Grefkes C., Shah N.J., Zilles K., Piza-Katzer H. (2008). Central adaptation following heterotopic hand replantation probed by fMRI and effective connectivity analysis. Exp. Neurol..

[bb0065] Feigin A., Ghilardi M.-F., Huang C., Ma Y., Carbon M., Guttman M. (2006). Preclinical Huntington's disease: compensatory brain responses during learning. Ann. Neurol..

[bb0075] Fox P.T., Friston K.J. (2012). Distributed processing; distributed functions?. Neuroimage.

[bb0080] Friston K. (2009). Causal modelling and brain connectivity in functional magnetic resonance imaging. PLoS Biol..

[bb0085] Friston K. (2012). Ten ironic rules for non-statistical reviewers. Neuroimage.

[bb0090] Friston K.J., Frith C.D., Turner R., Frackowiak R.S.J. (1995). Characterizing evoked hemodynamics with fMRI. Neuroimage.

[bb0095] Friston K.J., Harrison L., Penny W. (2003). Dynamic causal modelling. Neuroimage.

[bb0100] Gavazzi C., Nave R.D., Petralli R., Rocca M.A., Guerrini L., Tessa C. (2007). Combining functional and structural brain magnetic resonance imaging in Huntington disease. J. Comput. Assist. Tomogr..

[bb9005] Geyer S., Matelli M., Luppino G., Zilles K. (2000). Functional neuroanatomy of the primate isocortical motor system. Anatomy and Embryology.

[bb0105] Goebel R., Roebroeck A., Kim D.-S., Formisano E. (2003). Investigating directed cortical interactions in time-resolved fMRI data using vector autoregressive modeling and Granger causality mapping. Magn. Reson. Imaging.

[bb0110] Granger C.W.J. (1980). Testing for causality: a personal viewpoint. J. Econ. Dyn. Control..

[bb0120] Grefkes C., Nowak D.A., Eickhoff S.B., Dafotakis M., Küst J., Karbe H. (2008). Cortical connectivity after subcortical stroke assessed with functional magnetic resonance imaging. Ann. Neurol..

[bb0115] Grefkes C., Eickhoff S.B., Nowak D.A., Dafotakis M., Fink G.R. (2008). Dynamic intra- and interhemispheric interactions during unilateral and bilateral hand movements assessed with fMRI and DCM. Neuroimage.

[bb0125] Grefkes C., Nowak D.A., Wang L.E., Dafotakis M., Eickhoff S.B., Fink G.R. (2010). Modulating cortical connectivity in stroke patients by rTMS assessed with fMRI and dynamic causal modeling. Neuroimage.

[bb0130] Groppa S., Werner-Petroll N., Münchau A., Deuschl G., Ruschworth M.F.S., Siebner H.R. (2012). A novel dual-site transcranial magnetic stimulation paradigm to probe fast facilitatory inputs from ipsilateral dorsal premotor cortex to primary motor cortex. Neuroimage.

[bb0140] Holtzer R. (2009). Age effects on load-dependent brain activations in working memory for novel material. Brain Res..

[bb0145] Iacoboni M. (2006). Visuo-motor integration and control in the human posterior parietal cortex: evidence from TMS and fMRI. Neuropsychologia.

[bb0150] Kasess C.H., Windischberger C., Cunnington R., Lanzenberger R., Pezawas L., Moser E. (2008). The suppressive influence of SMA on M1 in motor imagery revealed by fMRI and dynamic causal modeling. Neuroimage.

[bb0155] Katzman R. (1993). Education and the prevalence of dementia and Alzheimer's disease. Neurology.

[bb0160] Kiebel S.J., Klöppel S., Weiskopf N., Friston K.J. (2007). Dynamic causal modeling: a generative model of slice timing in fMRI. Neuroimage.

[bb0165] Klöppel S., Draganski B., Golding C.V., Chu C., Nagy Z., Cook P.A. (2008). White matter connections reflect changes in voluntary-guided saccades in pre-symptomatic Huntington's disease. Brain.

[bb0170] Klöppel S., Draganski B., Siebner H.R., Tabrizi S.J., Weiller C., Frackowiak R.S.J. (2009). Functional compensation of motor function in pre-symptomatic Huntington's disease. Brain.

[bb0180] Koch G., Rothwell J.C. (2009). TMS investigations into the task-dependent functional interplay between human posterior parietal and motor cortex. Behav. Brain Res..

[bb0175] Koch G., Franca M., Olmo M.F.D., Cheeran B., Milton R., Sauco M.A. (2006). Time course of functional connectivity between dorsal premotor and contralateral motor cortex during movement selection. J. Neurosci..

[bb0185] Kötter R., Stephan K.E. (2003). Network participation indices: characterizing component roles for information processing in neural networks. Neural Netw..

[bb0190] Langbehn D.R., Brinkman R.R., Falush D., Paulsen J.S., Hayden M.R. (2004). A new model for prediction of the age of onset and penetrance for Huntington's disease based on CAG length. Clin. Genet..

[bb0195] Langbehn D.R., Hayden M.R., Paulsen J.S. (2010). CAG‐repeat length and the age of onset in Huntington disease (HD): a review and validation study of statistical approaches. Am. J. Med. Genet. B Neuropsychiatr. Genet..

[bb0200] Lee J.K., Mathews K., Schlaggar B., Perlmutter J., Paulsen J.S., Epping E. (2012). Measures of growth in children at risk for Huntington disease. Neurology.

[bb0205] Lehéricy S., Bardinet E., Tremblay L., Moortele P.-F.V. de, Pochon J.-B., Dormont D. (2006). Motor control in basal ganglia circuits using fMRI and brain atlas approaches. Cereb. Cortex.

[bb0210] Luppino G., Matelli M., Camarda R., Rizzolatti G. (1993). Corticocortical connections of area F3 (SMA-proper) and area F6 (pre-SMA) in the macaque monkey. J. Comp. Neurol..

[bb0215] Marder K., Mehler M.F. (2012). Development and neurodegeneration Turning HD pathogenesis on its head. Neurology.

[bb0220] Mars R.B., Jbabdi S., Sallet J., O'Reilly J.X., Croxson P.L., Olivier E. (2011). Diffusion-weighted imaging tractography-based parcellation of the human parietal cortex and comparison with human and macaque resting-state functional connectivity. J. Neurosci..

[bb0225] Matelli M., Luppino G. (2001). Parietofrontal circuits for action and space perception in the macaque monkey. Neuroimage.

[bb0230] McIntyre C.C., Hahn P.J. (2010). Network perspectives on the mechanisms of deep brain stimulation. Neurobiol. Dis..

[bb0235] Mühlau M., Gaser C., Wohlschläger A.M., Weindl A., Städtler M., Valet M. (2007). Striatal gray matter loss in Huntington's disease is leftward biased. Mov. Disord..

[bb0240] Murray A.D., Staff R.T., McNeil C.J., Salarirad S., Ahearn T.S., Mustafa N. (2011). The balance between cognitive reserve and brain imaging biomarkers of cerebrovascular and Alzheimer's diseases. Brain.

[bb0245] Nachev P., Kennard C., Husain M. (2008). Functional role of the supplementary and pre-supplementary motor areas. Nat. Rev. Neurosci..

[bb0250] Nakamura K., Sakai K., Hikosaka O. (1998). Neuronal activity in medial frontal cortex during learning of sequential procedures. J. Neurophysiol..

[bb0255] Narayana S., Laird A.R., Tandon N., Franklin C., Lancaster J.L., Fox P.T. (2012). Electrophysiological and functional connectivity of the human supplementary motor area. Neuroimage.

[bb0260] Nithianantharajah J., Hannan A.J. (2011). Mechanisms mediating brain and cognitive reserve: experience-dependent neuroprotection and functional compensation in animal models of neurodegenerative diseases. Prog. Neuro-Psychopharmacol. Biol. Psychiatry.

[bb0265] Nithianantharajah J., Barkus C., Vijiaratnam N., Clement O., Hannan A.J. (2009). Modeling brain reserve: experience-dependent neuronal plasticity in healthy and Huntington's disease transgenic mice. Am. J. Geriatr. Psychiatry.

[bb0270] Nopoulos P.C., Aylward E.H., Ross C.A., Mills J.A., Langbehn D.R., Johnson H.J. (2011). Smaller intracranial volume in prodromal Huntington's disease: evidence for abnormal neurodevelopment. Brain.

[bb0275] Novak M.J.U., Warren J.D., Henley S.M.D., Draganski B., Frackowiak R.S., Tabrizi S.J. (2012). Altered brain mechanisms of emotion processing in pre-manifest Huntington's disease. Brain.

[bb0280] O'Shea J., Johansen-Berg H., Trief D., Göbel S., Rushworth M.F.S. (2007). Functionally specific reorganization in human premotor cortex. Neuron.

[bb0285] O'Shea J., Sebastian C., Boorman E.D., Johansen-Berg H., Rushworth M.F.S. (2007). Functional specificity of human premotor–motor cortical interactions during action selection. Eur. J. Neurosci..

[bb0300] Rizzolatti G., Fogassi L., Gallese V. (1997). Parietal cortex: from sight to action. Curr. Opin. Neurobiol..

[bb0305] Rosas H.D., Hevelone N.D., Zaleta A.K., Greve D.N., Salat D.H., Fischl B. (2005). Regional cortical thinning in preclinical Huntington disease and its relationship to cognition. Neurology.

[bb0310] Rounis E., Lee L., Siebner H.R., Rowe J.B., Friston K.J., Rothwell J.C. (2005). Frequency specific changes in regional cerebral blood flow and motor system connectivity following rTMS to the primary motor cortex. Neuroimage.

[bb0315] Rowe J.B., Hughes L.E., Barker R.A., Owen A.M. (2010). Dynamic causal modelling of effective connectivity from fMRI: are results reproducible and sensitive to Parkinson's disease and its treatment?. Neuroimage.

[bb0325] Rushworth M.F., Johansen-Berg H., Göbel S., Devlin J. (2003). The left parietal and premotor cortices: motor attention and selection. Neuroimage.

[bb0320] Rushworth M.F.S., Walton M.E., Kennerley S.W., Bannerman D.M. (2004). Action sets and decisions in the medial frontal cortex. Trends Cogn. Sci..

[bb0330] Sarfeld A.-S., Diekhoff S., Wang L.E., Liuzzi G., Uludağ K., Eickhoff S.B. (2012). Convergence of human brain mapping tools: neuronavigated TMS parameters and fMRI activity in the hand motor area. Hum. Brain Mapp..

[bb0335] Satz P. (1993). Brain reserve capacity on symptom onset after brain injury: a formulation and review of evidence for threshold theory. Neuropsychology.

[bb0340] Scahill R.I., Hobbs N.Z., Say M.J., Bechtel N., Henley S.M.D., Hyare H., Langbehn D.R., Jones R., Leavitt B.R., Roos R.A.C., Durr A., Johnson H., Lehéricy S., Craufurd D., Kennard C., Hicks S.L., Stout J.C., Reilmann R., Tabrizi S.J., Investigators the T.-H. (2013). Clinical impairment in premanifest and early Huntington's disease is associated with regionally specific atrophy. Human Brain Mapping.

[bb0345] Schluter N.D., Rushworth M.F., Passingham R.E., Mills K.R. (1998). Temporary interference in human lateral premotor cortex suggests dominance for the selection of movements. A study using transcranial magnetic stimulation. Brain.

[bb0350] Seghier M.L., Zeidman P., Neufeld N.H., Leff A.P., Price C.J. (2010). Identifying abnormal connectivity in patients using dynamic causal modeling of fMRI responses. Front. Syst. Neurosci..

[bb0355] Steffener J., Reuben A., Rakitin B., Stern Y. (2011). Supporting performance in the face of age-related neural changes: testing mechanistic roles of cognitive reserve. Brain Imaging Behav..

[bb0360] Stephan K.E. (2004). On the role of general system theory for functional neuroimaging. J. Anat..

[bb0375] Stephan K.E., Weiskopf N., Drysdale P.M., Robinson P.A., Friston K.J. (2007). Comparing hemodynamic models with DCM. Neuroimage.

[bb0370] Stephan K.E., Penny W.D., Daunizeau J., Moran R.J., Friston K.J. (2009). Bayesian model selection for group studies. Neuroimage.

[bb0365] Stephan K.E., Penny W.D., Moran R.J., den Ouden H.E.M., Daunizeau J., Friston K.J. (2010). Ten simple rules for dynamic causal modeling. Neuroimage.

[bb0380] Stern Y. (2002). What is cognitive reserve? Theory and research application of the reserve concept. J. Int. Neuropsychol. Soc..

[bb0385] Stern Y. (2009). Cognitive reserve. Neuropsychologia.

[bb0390] Tabrizi S.J., Langbehn D.R., Leavitt B.R., Roos R.A., Durr A., Craufurd D. (2009). Biological and clinical manifestations of Huntington's disease in the longitudinal TRACK-HD study: cross-sectional analysis of baseline data. Lancet Neurol..

[bb0395] Tabrizi S.J., Scahill R.I., Durr A., Roos R.A., Leavitt B.R., Jones R. (2011). Biological and clinical changes in premanifest and early stage Huntington's disease in the TRACK-HD study: the 12-month longitudinal analysis. Lancet Neurol..

[bb0400] Tabrizi S.J., Reilmann R., Roos R.A., Durr A., Leavitt B., Owen G. (2012). Potential endpoints for clinical trials in premanifest and early Huntington's disease in the TRACK-HD study: analysis of 24 month observational data. Lancet Neurol..

[bb9010] Tanaka M., Watanabe Y. (2011). Neural compensation mechanisms to regulate motor output during physical fatigue. Brain Research.

[bb0405] The Huntington's Disease Collaborative Research Group (1993). A novel gene containing a trinucleotide repeat that is expanded and unstable on Huntington's disease chromosomes. Cell.

[bb0410] Valenzuela M.J. (2008). Brain reserve and the prevention of dementia. Curr. Opin. Psychiatry.

[bb0415] Walker F.O. (2007). Huntington's disease. Lancet.

[bb0420] Wang L.E., Fink G.R., Diekhoff S., Rehme A.K., Eickhoff S.B., Grefkes C. (2011). Noradrenergic enhancement improves motor network connectivity in stroke patients. Ann. Neurol..

[bb0425] Witt S.T., Laird A.R., Meyerand M.E. (2008). Functional neuroimaging correlates of finger-tapping task variations: an ALE meta-analysis. Neuroimage.

[bb0430] Wolf R.C., Vasic N., Schonfeldt-Lecuona C., Landwehrmeyer G.B., Ecker D. (2007). Dorsolateral prefrontal cortex dysfunction in presymptomatic Huntington's disease: evidence from event-related fMRI. Brain.

[bb0435] Wolf R.C., Sambataro F., Vasic N., Schönfeldt-Lecuona C., Ecker D., Landwehrmeyer B. (2008). Aberrant connectivity of lateral prefrontal networks in presymptomatic Huntington's disease. Exp. Neurol..

[bb0440] Wolf R.C., Sambataro F., Vasic N., Schönfeldt-Lecuona C., Ecker D., Landwehrmeyer B. (2008). Altered frontostriatal coupling in pre-manifest Huntington's disease: effects of increasing cognitive load. Eur. J. Neurol..

[bb0455] Wolf R.C., Grön G., Sambataro F., Vasic N., Wolf N.D., Thomann P.A. (2012). Brain activation and functional connectivity in premanifest Huntington's disease during states of intrinsic and phasic alertness. Hum. Brain Mapp..

[bb0460] Wolf R.C., Sambataro F., Vasic N., Wolf N.D., Thomann P.A., Saft C. (2012). Default-mode network changes in preclinical Huntington's disease. Exp. Neurol..

[bb0465] Wood N.I., Glynn D., Morton A.J. (2011). “Brain training” improves cognitive performance and survival in a transgenic mouse model of Huntington's disease. Neurobiol. Dis..

[bb0470] Zeki S., Shipp S. (1988). The functional logic of cortical connections. Nature.

