# Supplementary material for: Interregional compensatory mechanisms of motor functioning in progressing preclinical neurodegeneration
Source: Neuroimage. 2013 Jul 15;75:146–54. doi: 10.1016/j.neuroimage.2013.02.058 (PMC3899022; doi:10.1016/j.neuroimage.2013.02.058)
Supplement: Inline Supplementary Table S2 [file mmc1.doc]

**Supplement 1. Dynamic Causal Modelling: Motivation of Model Specification**


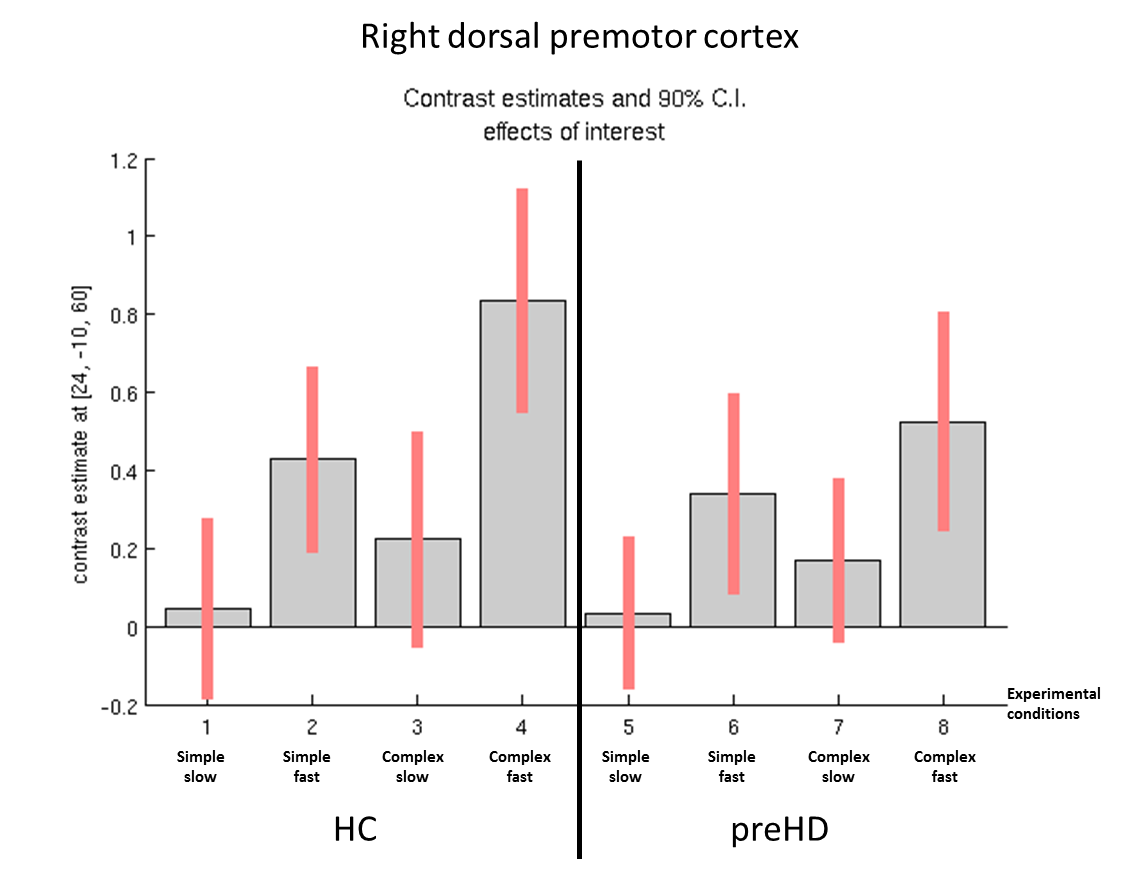

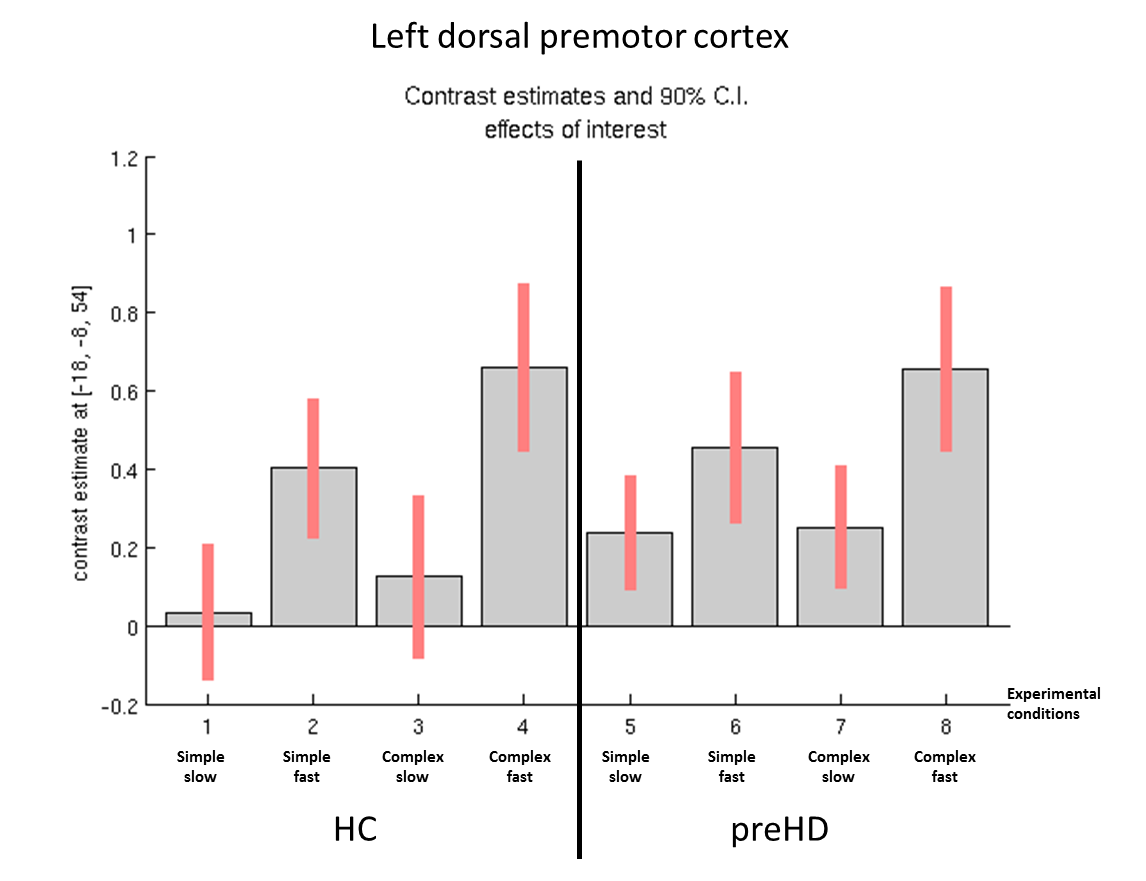
As mentioned in the main text, we chose a model specification based on a former GLM analyses (Klöppel et al., 2009) with additional interrogations regarding the rPMd and lM1. The pattern of activation in rPMd, which led to the inclusion of modulations by speed and complexity in the DCM, is depicted below. Interestingly, there was more activation during the complex fast condition in HC compared to preHD (p=0.02). For comparison, the activation pattern in left PMd is displayed as well where no condition-specific between-group differences were observed.

It is important to stress that the model we chose to investigate is only one of numerous possibilities to capture the data (Friston et al., 2011) and is always a coarse simplification. Intuitively, one might argue that a more complex model taking to account every single previous GLM result would be a better explanation of the data. However, there is an accuracy-complexity tradeoff in the estimation process of DCMs (Friston et al., 2003), which results in the rejection of a model that might be more accurate in describing the data (accuracy), but is – compared to another model – too complex and might overfit the data. Thus, the aim is to find the most parsimonious as well as accurate model.

To validate the model used in this study (Model 1), we specified a second model (Model 2) including all former GLM effects (Klöppel et al., 2009), which resulted in 82 condition-independent and modulatory parameters (compared with 65 parameters in our more parsimonious model). We compared models using a random effects Bayesian Model Selection (BMS) procedure (Stephan et al., 2009) with the two models in both preHD and HC.

The clear winning model of BMS was Model 1 with an exceedance probability of 0.99 in preHD as well as HC and an exceedance probability of 1 across both groups. The exceedance probability used in random effects BMS can be interpreted as the belief that a certain model is more likely than any other model given the data (Stephan et al., 2009).

References

Daunizeau J, David O, Stephan KE. Dynamic causal modelling: A critical review of the biophysical and statistical foundations. NeuroImage. 2011 Sep 15;58(2):312–22.

Friston KJ, Harrison L, Penny W. Dynamic causal modelling. NeuroImage. 2003 Aug;19(4):1273–302.

Friston KJ, Li B, Daunizeau J, Stephan KE. Network discovery with DCM. NeuroImage. 2011 Jun;56(3):1202–21.

Klöppel S, Draganski B, Siebner HR, Tabrizi SJ, Weiller C, Frackowiak RSJ. Functional Compensation of Motor Function in Pre-Symptomatic Huntington’s Disease. Brain. 2009 Jun 1;132(6):1624–32.

Stephan KE, Penny WD, Daunizeau J, Moran RJ, Friston KJ. Bayesian model selection for group studies. NeuroImage. 2009 Jul;46(4):1004–17.

Stephan KE, Penny WD, Moran RJ, den Ouden HEM, Daunizeau J, Friston KJ. Ten simple rules for dynamic causal modeling. NeuroImage. 2010 Feb;49(4):3099–109.
