# Supplementary material for: Interregional compensatory mechanisms of motor functioning in progressing preclinical neurodegeneration
Source: Neuroimage. 2013 Jul 15;75:146–54. doi: 10.1016/j.neuroimage.2013.02.058 (PMC3899022; doi:10.1016/j.neuroimage.2013.02.058)
Supplement: Inline Supplementary Table S3 [file mmc2.doc]

**Supplementary Table S2.**

**MNI coordinates of individual peak voxel in each participant for volume of interest extraction
(preHD N = 15; HC N = 12)**

|  | **pSMA** | | | **cSMA** | | | **lM1** | | | **lPMd** | | | **lSPC** | | | **rSPC** | | | **rPMd** | | |
| --- | --- | --- | --- | --- | --- | --- | --- | --- | --- | --- | --- | --- | --- | --- | --- | --- | --- | --- | --- | --- | --- |
|  | x | y | z | x | y | z | x | y | z | x | y | z | x | y | z | x | y | z | x | y | z |
| **preHD** |  |  |  |  |  |  |  |  |  |  |  |  |  |  |  |  |  |  |  |  |  |
| 1 | 0 | 12 | 60 | -6 | -16 | 62 | -48 | -20 | 56 | -26 | -8 | 60 | -20 | -66 | 54 | 24 | -66 | 62 | 22 | -6 | 62 |
| 2 | 4 | 2 | 52 | -2 | -10 | 56 | -34 | -16 | 66 | -24 | -8 | 56 | -22 | -60 | 62 | 24 | -54 | 68 | 26 | -8 | 64 |
| 3 | -2 | 6 | 46 | -4 | -8 | 56 | -38 | -26 | 58 | -28 | -8 | 54 | -32 | -58 | 50 | 30 | -60 | 64 | 26 | -2 | 62 |
| 4 | -6 | 8 | 50 | 0 | -10 | 62 | -42 | -20 | 58 | -22 | -12 | 62 | -26 | -62 | 64 | 18 | -62 | 64 | 24 | -6 | 56 |
| 5 | -8 | 8 | 52 | -12 | -6 | 60 | -38 | -26 | 64 | -26 | -2 | 54 | -28 | -54 | 52 | 28 | -64 | 54 | 28 | -6 | 58 |
| 6 | 4 | 12 | 58 | -2 | -12 | 54 | -36 | -24 | 60 | -24 | -4 | 60 | -18 | -58 | 64 | 20 | -62 | 66 | 32 | -6 | 56 |
| 7 | 10 | 6 | 54 | -2 | -18 | 52 | -42 | -10 | 56 | -26 | -8 | 50 | -22 | -58 | 60 | 30 | -62 | 62 | 26 | -8 | 68 |
| 8 | -4 | 14 | 56 | -4 | -10 | 62 | -44 | -26 | 58 | -26 | -14 | 54 | -32 | -58 | 62 | 26 | -62 | 62 | 30 | -4 | 56 |
| 9 | 0 | 10 | 62 | -4 | -10 | 58 | -48 | -14 | 56 | -14 | -4 | 62 | -28 | -54 | 64 | 20 | -62 | 62 | 32 | -4 | 60 |
| 10 | 0 | -2 | 58 | -6 | -18 | 52 | -42 | -26 | 64 | -28 | -8 | 54 | -24 | -54 | 52 | 30 | -58 | 62 | 28 | -6 | 68 |
| 11 | 4 | 10 | 58 | 0 | -14 | 54 | -44 | -18 | 44 | -26 | -14 | 54 | -22 | -54 | 64 | 18 | -58 | 54 | 30 | -16 | 64 |
| 12 | 4 | 10 | 50 | -6 | -10 | 58 | -38 | -22 | 62 | -22 | -16 | 52 | -32 | -60 | 54 | 32 | -60 | 60 | 28 | -18 | 64 |
| 13 | 0 | 14 | 48 | -2 | -14 | 62 | -44 | -20 | 58 | -22 | -16 | 56 | -14 | -58 | 56 | 22 | -64 | 54 | 32 | -6 | 60 |
| 14 | 2 | 14 | 50 | -2 | -6 | 48 | -38 | -20 | 68 | -24 | -12 | 58 | -16 | -62 | 60 | 14 | -60 | 66 | 26 | -14 | 54 |
| 15 | 4 | 14 | 50 | -6 | -8 | 62 | -40 | -22 | 64 | -24 | 0 | 54 | -30 | -52 | 60 | 30 | -52 | 60 | 18 | -16 | 64 |
| **HC** | x | y | z | x | y | z | x | y | z | x | y | z | x | y | z | x | y | z | x | y | z |
| 1 | 6 | 2 | 56 | 0 | -12 | 54 | -38 | -22 | 62 | -14 | -6 | 62 | -24 | -64 | 56 | 22 | -62 | 64 | 28 | -8 | 64 |
| 2 | -8 | 4 | 46 | -14 | -14 | 56 | -38 | -22 | 56 | -24 | -8 | 60 | -24 | -62 | 64 | 22 | -62 | 58 | 28 | -12 | 58 |
| 3 | 8 | 10 | 56 | -6 | -8 | 58 | -42 | -12 | 58 | -22 | -12 | 58 | -20 | -56 | 64 | 26 | -52 | 68 | 18 | -8 | 64 |
| 4 | 0 | 14 | 18 | 0 | -16 | 56 | -38 | -26 | 64 | -18 | 0 | 54 | -22 | -60 | 60 | 22 | -64 | 62 | 28 | -16 | 56 |
| 5 | 2 | 10 | 60 | -6 | -6 | 54 | -38 | -26 | 60 | -24 | -8 | 62 | -32 | -54 | 60 | 30 | -64 | 58 | 28 | -4 | 64 |
| 6 | 0 | 8 | 50 | -2 | -18 | 58 | -40 | -14 | 66 | -22 | 2 | 46 | -24 | -58 | 66 | 28 | -54 | 68 | 28 | -18 | 64 |
| 7 | 0 | 4 | 54 | -2 | -18 | 50 | -38 | -12 | 58 | -24 | -6 | 52 | -24 | -64 | 60 | 22 | -66 | 58 | 24 | -4 | 52 |
| 8 | -4 | 10 | 54 | -2 | -10 | 56 | -38 | -18 | 60 | -26 | -8 | 58 | -24 | -54 | -64 | 32 | -60 | 60 | 26 | -2 | 68 |
| 9 | 8 | 6 | 56 | -2 | -10 | 52 | -40 | -22 | 62 | -26 | -8 | 58 | -20 | -62 | 64 | 26 | -52 | 68 | 28 | -6 | 68 |
| 10 | 0 | 10 | 50 | 0 | -6 | 52 | -36 | -24 | 62 | -18 | 0 | 60 | -26 | -58 | 62 | 18 | -60 | 58 | 30 | -8 | 60 |
| 11 | -4 | 12 | 48 | -4 | -14 | 50 | -46 | -22 | 54 | -12 | -6 | 60 | -24 | -66 | 52 | 28 | -58 | 64 | 30 | -10 | 68 |
| 12 | -4 | 10 | 50 | -8 | -6 | 62 | -40 | -8 | 60 | -26 | -10 | 58 | -22 | -64 | 56 | 26 | -64 | 64 | 30 | -10 | 66 |
