# Supplementary material for: Interregional compensatory mechanisms of motor functioning in progressing preclinical neurodegeneration
Source: Neuroimage. 2013 Jul 15;75:146–54. doi: 10.1016/j.neuroimage.2013.02.058 (PMC3899022; doi:10.1016/j.neuroimage.2013.02.058)
Supplement: Inline Supplementary Table S4 [file mmc3.doc]

**Supplementary Table S3.**

Descriptive statistics of fixed and modulatory DCM parameter estimates and results from Wilcoxon signed rank tests to test whether the parameter is significantly different from zero within groups.

|  | **preHD** | | | | **HC** | | |
| --- | --- | --- | --- | --- | --- | --- | --- |
| **DCM parameter** | **N** | **Minimum** | **Maximum** | **Median** | **Minimum** | **Maximum** | **Median** |
| pSMA🡪pSMA, F | 12 | -1.01 | -.83 | -.97** | -1.0 | -.87 | -.96** |
| cSMA🡪pSMA, F | 12 | -.25 | .19 | -.01 | -.18 | .21 | .03 |
| lPMd🡪pSMA, F | 12 | -.23 | .15 | .02 | -.09 | .23 | .05 |
| lSPC🡪pSMA, F | 12 | -.12 | .44 | .13** | -.085 | .59 | .24** |
| rSPC🡪pSMA, F | 12 | .01 | .79 | .19 | -.58 | .43 | .22 |
| rPMd🡪pSMA, F | 12 | -.04 | .20 | .01 | -.16 | .35 | .02 |
| pSMA🡪cSMA. F | 12 | -.40 | .65 | .08* | -.12 | .95 | .21* |
| cSMA🡪cSMA, F | 12 | -1.00 | -.84 | -.95** | -1.04 | -.82 | -.98** |
| lM1🡪cSMA, F | 12 | -.02 | .38 | .12** | -.11 | .23 | .03 |
| lPMd🡪cSMA, F | 12 | -.15 | .75 | .35** | -.08 | .80 | .29** |
| cSMA🡪lM1, F | 12 | .02 | .48 | .21** | -.25 | .92 | .18** |
| lM1🡪lM1, F | 12 | -1.02 | -.83 | -.98** | -1.03 | -.73 | -.96** |
| lPMd🡪lM1, F | 12 | .02 | 1.02 | .64** | .17 | .93 | .63** |
| pSMA🡪lPMd, F | 12 | -.29 | .26 | .00 | -.20 | .40 | .05 |
| cSMA🡪lPMd, F | 12 | -.23 | .20 | .03 | -.18 | .17 | -.03 |
| lM1🡪lPMd, F | 12 | -.26 | .11 | .02 | -.20 | .13 | -.04 |
| lPMd🡪lPMd, F | 12 | -1.07 | -.82 | -.96** | -1.04 | -.83 | -.95** |
| lSPC🡪lPMd, F | 12 | .00 | .84 | .27** | -.21 | .85 | .33** |
| rSPC🡪lPMd, F | 12 | -.07 | .88 | .19** | -.15 | .58 | .29** |
| rPMd🡪lPMd, F | 12 | .02 | .26 | .10** | -.19 | .61 | .07 |
| pSMA🡪lSPC, F | 12 | -.16 | .43 | -.04 | -.33 | .00 | -.12** |
| lPMd🡪lSPC, F | 12 | -.36 | .20 | -.06 | -.36 | .04 | -.20** |
| lSPC🡪lSPC, F | 12 | -1.03 | -.97 | -.99** | -1.06 | -.93 | -1.01** |
| rSPC🡪lSPC, F | 12 | -.02 | .16 | .05** | -.14 | .18 | .04 |
| rPMd🡪 lSPC, F | 12 | -.38 | .14 | -.04 | -.37 | .03 | -.13* |
| pSMA🡪rSPC, F | 12 | -.35 | .24 | -.06 | -.27 | .12 | -.11* |
| lPMd🡪rSPC, F | 12 | -.53 | .36 | -.11 | -.39 | .29 | -.06* |
| lSPC🡪rSPC, F | 12 | -.11 | .20 | -.00 | -.14 | .12 | .00 |
| rSPC🡪rSPC, F | 12 | -1.05 | -.88 | -.99** | -1.03 | -.91 | -.98** |
| rPMd🡪rSPC, F | 12 | -.32 | .20 | -.07 | -.32 | .05 | -.15** |
|  | **preHD** | | | | **HC** | | |
| **DCM parameter** | **N** | **Minimum** | **Maximum** | **Median** | **Minimum** | **Maximum** | **Median** |
| pSMA🡪rPMd, F | 12 | -.19 | .24 | .01 | -.27 | .35 | .07 |
| lPMd🡪rPMd, F | 12 | -.06 | .18 | .04** | -.05 | .27 | .05* |
| lSPC🡪rPMd, F | 12 | -.17 | .33 | .22* | -.15 | .38 | .26** |
| rSPC🡪rPMd, F | 12 | -.00 | .44 | .18** | -.07 | .51 | .19* |
| rPMd🡪rPMd, F | 12 | -1.01 | -.94 | -.98** | -1.00 | -.83 | -.97** |
| cSMA🡪pSMA, C | 12 | -.64 | .73 | .08 | -1.12 | .23 | -.29* |
| lPMd🡪pSMA, C | 12 | -.13 | .30 | .08* | -.67 | .58 | -.07 |
| lSPC🡪pSMA, C | 12 | -.46 | .99 | .11 | -.69 | .91 | .13 |
| rSPC🡪pSMA, C | 12 | -.26 | 1.09 | .19 | -1.16 | .76 | .22 |
| rPMd🡪pSMA, C | 12 | -.44 | .77 | .19* | -.69 | .60 | -.01 |
| lPMd🡪lM1, C | 12 | -1.85 | 1.08 | -.57* | -1.60 | 1.21 | -.23 |
| pSMA🡪lSPC, C | 12 | -2.2 | .52 | -.03 | -.35 | 1.10 | .18* |
| lPM🡪lSPC, C | 12 | -.33 | .66 | .01 | -.37 | .70 | .02 |
| rSPC🡪lSPC, C | 12 | -.55 | .41 | .09 | -.04 | 1.37 | .24** |
| rPMd🡪lSPC, C | 12 | -.78 | .51 | -.01 | -.80 | .52 | -.12 |
| pSMA🡪rSPC, C | 12 | -1.02 | 1.04 | .01 | -.70 | .80 | .18 |
| lPMd🡪rSPC, C | 12 | -.59 | 1.05 | .03 | -.75 | .74 | .03 |
| lSPC🡪rSPC, C | 12 | -.14 | 1.02 | .08 | -.35 | .36 | .24 |
| rPMd🡪rSPC, C | 12 | -.91 | .49 | .02 | -.78 | .22 | -.02 |
| pSMA🡪rPMd, C | 12 | -.51 | .65 | .02 | -.44 | .28 | -.02 |
| lPMd🡪rPMd, C | 12 | -.37 | .33 | -.07 | -.49 | .41 | .09 |
| lSPC🡪rPMd, C | 12 | -1.22 | .40 | -.07 | -.49 | 1.09 | .04 |
| rSPC🡪rPMd, C | 12 | -.33 | 1.23 | .09 | -.45 | .52 | .01 |
| pSMA🡪cSMA, S | 12 | -1.06 | .92 | .00 | -1.31 | 2.30 | .42* |
| lM1🡪cSMA, S | 12 | -.58 | .48 | .01 | -.69 | .40 | .05 |
| lPMd🡪cSMA, S | 12 | -.13 | 1.12 | .22* | -.27 | 1.11 | .48** |
| cSMA🡪M1, S | 12 | -.30 | 1.87 | .65** | -.00 | 2.32 | .62** |
| pSMA🡪rSPC, S | 12 | -1.69 | .35 | -.01 | -.90 | 1.37 | -.02 |
| lPMd🡪rSPC, S | 12 | -.37 | .28 | -.01 | -.77 | .27 | -.07 |
| lSPC🡪rSPC, S | 12 | -.16 | .49 | .10 | -.23 | .62 | .28** |
| rPMd🡪rSPC, S | 12 | -.37 | .30 | .02 | -.52 | 1.26 | -.21 |
| pSMA🡪rPMd, S | 12 | -.45 | .48 | .01 | -.56 | .83 | .07 |
| lPMd🡪rPMd, S | 12 | -.57 | .41 | .00 | -.46 | .81 | .11 |
| lSPC🡪rPMd, S | 12 | -.31 | .72 | .28* | -.55 | .28 | .19 |
| rSPC🡪rPMd, S | 12 | -.47 | .63 | .02 | -.33 | 1.20 | .35** |

Note: S = Speed (experimental modulation), C = complexity (experimental modulation), F = Fixed/condition independent

connection

Significance thresholds in Wilcoxon signed rank tests: * p < .05; ** p < .01;
