# Supplementary material for: Interregional compensatory mechanisms of motor functioning in progressing preclinical neurodegeneration
Source: Neuroimage. 2013 Jul 15;75:146–54. doi: 10.1016/j.neuroimage.2013.02.058 (PMC3899022; doi:10.1016/j.neuroimage.2013.02.058)
Supplement: Supplement 1 — Dynamic Causal Modelling: motivation of model specification. [file mmc4.doc]

**Supplementary Table S4.**

Rotated component matrix of the PCA with fixed and modulatory DCM parameters of the preHD group with eigenvalues and percentage of explained variance.

| **DCM parameters** | **Component with loadings** | | | | | | | | | |
| --- | --- | --- | --- | --- | --- | --- | --- | --- | --- | --- |
|  | **1** | **2** | **3** | **4** | **5** | **6** | **7** | **8** | **9** | **10** |
| rPMd🡪rSPC,S | **.852** |  |  | .321 |  | .238 |  | -.322 |  |  |
| lSPC🡪rSPC,S | **-.849** | .166 |  | -.286 |  | -.287 |  | -.188 | .105 | -.112 |
| rPMd🡪rSPC,C | **.844** | -.122 | -.153 | .328 |  |  | -.146 |  | .168 | .281 |
| rSPC🡪pSMA,C | **-.842** |  |  | .112 | -.172 | .141 | -.292 | .194 |  | .242 |
| lPMd🡪lSPC,C | **.792** | -.307 | -.185 | -.340 |  | .240 |  | .230 |  |  |
| lSPC🡪rSPC,C | **-.701** | -.173 | -.119 | -.521 |  |  |  | -.109 | -.380 | -.132 |
| lPMd🡪rPMd, F | **.674** | -.284 | .343 |  | .297 | .338 |  | -.253 |  | .269 |
| lPMd🡪cSMA, F | **.633** |  |  |  |  | .344 | .433 |  | .135 | .508 |
| rPMd🡪lPMd, F | **-.631** | -.211 | -.230 |  | -.382 | .555 |  | -.169 |  |  |
| rSPC🡪rPMd,C | **-.610** | .168 | -.371 | .558 | -.160 | .208 |  | -.208 |  |  |
| lPMd🡪rSPC,S | **.602** | .399 | .167 | -.122 | .363 | -.173 | -.278 | .108 | .402 |  |
| rPMd🡪lSPC,S | **.549** |  | .338 | .121 | .125 | -.247 | .322 | -.378 | -.155 | .407 |
| pSMA🡪 rSPC,C | .180 | **.906** | .279 | .239 |  |  |  |  |  |  |
| pSMA🡪lSPC,C |  | **.886** |  |  | -.254 | -.335 | -.115 |  |  |  |
| pSMA🡪rSPC,S |  | **.874** | .224 |  | -.229 | -.317 | -.141 |  |  |  |
| pSMA🡪rPMd,S | -.233 | **.817** | .228 | -.239 | .142 | .169 | -.119 | .222 | -.110 | -.210 |
| pSMA🡪cSMA,S | -.412 | **.795** | .184 | .252 |  |  |  | -.209 |  | -.187 |
| pSMA🡪cSMA, F | .292 | **.666** | -.168 | .585 | -.196 |  |  |  |  | .200 |
| pSMA🡪lSPC, F | .403 | **-.549** | .342 |  | .381 | .506 |  |  |  | -.123 |
| lPMd🡪rSPC, F | .166 |  | **.945** | -.223 |  | -.111 |  |  |  |  |
| lPMd🡪lSPC, F |  | .226 | **.933** |  | -.151 | -.120 |  |  |  | -.133 |
| rPMd🡪rSPC, F | -.222 | .352 | **.874** |  | -.182 |  |  |  |  | -.166 |
| rPMd🡪lSPC, F |  | .394 | **.792** | -.206 | -.241 | -.141 | -.118 |  | .130 | -.246 |
| pSMA🡪rSPC, F | .537 |  | **.733** | .366 |  |  |  |  |  | .133 |
| **DCM parameters** | **Component with loadings** | | | | | | | | | |
|  | **1** | **2** | **3** | **4** | **5** | **6** | **7** | **8** | **9** | **10** |
| pSMA🡪lPMd, F | -.174 | .528 | **.728** | -.105 | -.208 |  | -.187 |  | -.262 |  |
| lSPC🡪rSPC, F | .471 | -.113 | **.656** |  | .179 | .254 | .417 |  |  | .193 |
| lSPC-lSPC, F |  | -.452 | **.583** | .505 |  |  | .238 | -.266 | -.245 |  |
| rPMd🡪pSMA,C | -.397 | -.166 | **-.521** | .381 | .324 | .480 | .202 | .122 |  |  |
| lPMd🡪pSMA,C | -.221 | .107 |  | **-.932** | -.168 |  |  |  |  | .133 |
| rPMd🡪pSMA, F | .292 | .189 | -.135 | **.880** | -.204 |  | -.177 |  |  |  |
| lPMd🡪rPMd,S |  | -.516 | -.165 | **-.785** | -.144 | .109 |  | .206 |  | -.116 |
| pSMA-pSMA, F |  |  | -.474 | **.633** | -.130 | .300 |  | -.486 |  |  |
| rSPC🡪pSMA, F | -.341 | .414 | -.339 | **.627** | -.103 | .231 | -.214 | -.183 |  | .102 |
| cSMA🡪pSMA, F |  | -.168 | .135 | **-.621** | -.591 | -.332 | -.273 | .117 | .121 |  |
| rSPC🡪rPMd,S |  | -.206 | -.290 | **-.553** | .305 | -.142 | .164 | .374 | .417 | .268 |
| lPMd🡪rPMd,C | -.145 |  |  | -.208 | **-.875** |  | -.257 | .168 | -.116 | -.227 |
| rPMd🡪pSMA, F | .117 |  | .366 | .124 | **-.872** |  | -.148 | -.136 |  | .147 |
| rPMd🡪lSPC,C | .146 | -.113 | -.264 |  | **.770** |  | -.190 | .469 | .191 |  |
| lPMd🡪pSMA, F |  | .405 | .233 | .277 | **-.725** | -.142 | .199 | .240 | .238 |  |
| lPMd🡪rSPC,C | .446 | -.304 | -.237 | .111 | **.701** | .316 | -.105 | .125 | .113 |  |
| rSPC-rSPC, F | .177 | .272 | .399 |  | **.618** | .399 | .114 | .342 | .246 |  |
| lSPC🡪rPMd, F |  | -.157 |  | .570 | **.596** |  | -.156 |  | .269 | .420 |
| rPMd-rPMd, F |  | -.266 |  |  | .232 | **.905** |  |  | .181 |  |
| cSMA-cSMA, F | .162 | -.272 | -.109 |  | .124 | **.879** | .250 | -.183 |  |  |
| cSMA🡪lM1, F | -.190 | .260 | -.307 | .313 | -.232 | **.723** |  | -.155 | .268 | .136 |
| lSPC🡪pSMA,C | -.267 |  | .270 | -.216 | .275 | **-.659** | .280 | -.263 |  | -.228 |
| lM1🡪cSMA, F | .469 | .191 | .213 |  | .159 | **.655** | .263 |  | .380 | .155 |
| pSMA🡪rPMd,C | -.305 | .500 |  | .435 | .346 | **.514** |  |  |  | -.260 |
| rSPC🡪lSPC, F | .354 | -.322 | .269 |  | .319 | **.495** | -.206 | .488 | -.135 |  |
| rSPC🡪lSPC,C | -.299 | -.185 |  |  | -.107 |  | **.904** | -.145 |  | .106 |
| lM1🡪cSMA,S | -.175 | -.111 | -.486 | .330 |  |  | **-.747** | .105 | .145 |  |
| cSMA🡪lPMd, F | -.308 | .446 | .218 |  | -.215 | -.155 | **-.673** |  | -.340 |  |
| lPMd-lPMd, F |  | -.527 | -.143 |  |  | .409 | **.572** | -.408 | .149 | .107 |
| **DCM parameters** | **Component with loadings** | | | | | | | | | |
|  | **1** | **2** | **3** | **4** | **5** | **6** | **7** | **8** | **9** | **10** |
| lM1🡪lPMd, F | -.322 | .326 | .380 |  | -.389 | -.360 | **-.560** |  | -.188 |  |
| lSPC🡪pSMA, F |  | .230 | -.145 | -.181 |  |  |  | **.838** |  |  |
| cSMA🡪pSMA,C | .210 | -.230 |  | -.316 | .493 | .328 | -.122 | **.639** |  | .137 |
| lPMd🡪lM1,C | -.409 |  | .134 | -.279 |  |  |  | **.613** | -.112 | -.570 |
| lPMd🡪cSMA,S |  | -.498 |  | -.106 | .573 | .163 | .135 | **-.594** | .108 |  |
| rSPC🡪rPMd, F |  |  | .185 | -.101 | .343 |  |  | -.188 | **.808** |  |
| lM1-lM1, F |  |  |  |  | -.531 | .319 |  | -.218 | **.718** | .146 |
| rSPC🡪lPMd, F |  | .331 | -.341 | .160 |  | .275 | .309 |  | **.693** | -.227 |
| lSPC🡪rPMd,C |  | .171 |  | -.388 | .175 | -.351 | .243 | -.223 | **-.677** | -.290 |
| lSPC🡪lPMd, F | .324 | -.597 | -.172 | -.140 | .132 |  | .219 |  |  | **.629** |
| lPMd🡪lM1, F | .262 |  | -.436 |  | .250 | .237 | .288 | -.273 | .330 | **.597** |
| cSMA🡪lM1,S | .333 | -.408 | -.380 | .439 |  | .373 |  |  |  | **.448** |
| Eigenvalues before rotation (after rotation) | 16.22 (9.54) | 10.28 (8.94) | 9.90 (8.47) | 6.44 (7.51) | 5.81  (7.27) | 4.85 (6.81) | 4.13 (4.34) | 2.78 (4.33) | 1.96 (3.82) | 1.68  (3.05) |
| % variance explained before rotation  (after rotation) | 24.96  (14.67) | 15.82 (13.76) | 15.24 (13.03) | 9.91 (11.56) | 8.93 (11.19) | 7.45 (10.48) | 6.36 (6.68) | 4.27 (6.66) | 3.02 (5.87) | 2.59 (4.68) |

Loadings of DCM parameters belonging to the respective component (columns) are printed in bold.
